# Structure-Dependent Antioxidant Activity of Ibogalogs: Impact of Methoxy Group Position on the Protective Activity in Model and Synaptosomal Lipid Membranes

**DOI:** 10.1021/acschemneuro.6c00087

**Published:** 2026-03-17

**Authors:** Paulina Kazmierska-Grebowska, Jacek Grebowski, Michał Żebrowski, Gino A. DiLabio, Oskar Ciesielski, Aneta Balcerczyk, Hugo R. Arias, Grzegorz Litwinienko

**Affiliations:** † Department of Neurobiology, Faculty of Biology and Environmental Protection, 196812 University of Lodz, Pomorska Str. 141/143, Lodz 90-236, Poland; ‡ Department of Oncobiology and Epigenetics, Faculty of Biology and Environmental Protection, University of Lodz, Pomorska Str.141/143 90-236, Lodz, Poland; § Military Institute of Medicine - National Research Institute, Szaserow Str.128, Warsaw 04-141, Poland; ∥ Faculty of Chemistry, 49605 University of Warsaw, Pasteura Str.1, Warsaw 02-093, Poland; ⊥ Department of Chemistry, The University of British Columbia, 3247 University Way, Kelowna, British Columbia V1 V 1 V7, Canada; # Department of Pharmacology and Physiology, Oklahoma State University, College of Osteopathic Medicine, Tahlequah, Oklahoma 74464, United States

**Keywords:** ibogalogs, antioxidants, lipid peroxidation, hemolysis, synaptosomes, bond strength

## Abstract

Indole-tetrahydroazepine derivatives of iboga alkaloids (ibogalogs) exhibit antidepressant, anxiolytic, promnesic, and antineuropathic effects, mainly via serotonergic targets. However, their physicochemical properties relevant to redox biology, including antioxidant activity and membrane stability, have remained poorly characterized. We investigated the antioxidant properties of three ibogalogs: ibogaminalog (DM506), ibogainalog (IBG), and tabernanthalog (TBG), using model lipid membranes (liposomes), human erythrocytes, and rat hippocampal and cortical synaptosomes exposed to AAPH-induced oxidative stress. Physicochemical descriptors, bond dissociation enthalpies, and ionization potentials were also calculated to assess membrane interactions and the antioxidant potential. All ibogalogs protected erythrocytes by reducing hemolysis, potassium efflux, and malondialdehyde levels, with the strongest effects observed for TBG; none induced hemolysis or K^+^ efflux at 0.01–10 μM. Ibogalogs also decreased lipid peroxidation in rat hippocampal and cortical synaptosomes. In liposomal systems, TBG showed the highest efficacy against lipid-peroxyl-radical-induced peroxidation, whereas DM506 and IBG mainly slowed autoxidation. Theoretical analysis indicated that the methoxy group substitution critically influences bond dissociation enthalpies, radical delocalization, and antioxidant potency. This first physicochemical characterization of antioxidant properties of ibogalogs enhances the understanding of their membrane-protective actions, complementing their neuromodulatory profiles.

## Graphical abstract

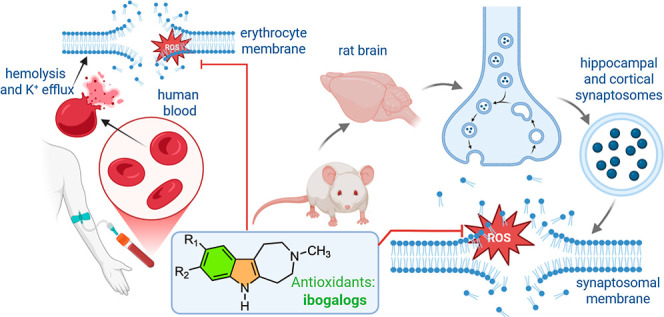

## 1 Introduction

Oxidative stress plays a central role in the pathogenesis of neurotoxicity,^1^ primarily due to the vulnerability of neuronal membranes to lipid peroxidation caused by reactive oxygen species (ROS).^2,3^ As membrane integrity is critical for proper neuronal function, maintaining effective antioxidant defense is essential for preserving neural homeostasis.^3–5^ Indole-hexahydroazepine derivatives of iboga alkaloids, known as “ibogalogs”, exhibit a wide range of neuromodulatory activities in rodents, including antidepressant, anxiolytic, promnesic, antiaddictive, and antineuropathic pain effects.^6–14^ Although these findings suggest neuromodulatory potential, the physicochemical basis of their activity, particularly in the context of oxidative stress, remains poorly characterized.^14,15^ Already published studies have demonstrated their affinity and functional activity at multiple serotonin (5-HT) receptor subtypes (including 5-HT_2A_, 5-HT_2B_, 5-HT_2C_, 5-HT_6_, and 5-HT_7_) as well as modulatory effects on monoamine transporters (SERT, NET, and DAT) and monoamine oxidases (MAO-A/B).^6–9,16,17^ However, most of their behavioral effects have been attributed to their interactions with metabotropic serotonin receptors, particularly the 5-HT_2A_ receptor subtype.^6,7,10–13^ Nevertheless, their impact on antioxidant defense mechanisms has remained largely unexplored. Given that ibogalogs preserve part of the iboga alkaloid structure, and considering that the latter have demonstrated protective effects in erythrocytes under oxidative stress,^14,18^ it is plausible that ibogalogs also possess redox-modulating properties relevant to membrane stability.

Lipid peroxidation is a key process in oxidative damage,^19^ especially in neuronal tissue, where the high content of polyunsaturated fatty acids makes lipid membranes susceptible to peroxyl radical-induced oxidation.^20^ Alterations in brain lipidome characteristics, including lipid concentration and composition, have been implicated in the onset and progression of neurodegenerative diseases.^21,22^ The investigation of antioxidant defense mechanisms against lipid peroxidation is, therefore, crucial for understanding how to protect neuronal cells from oxidative damage. In this context, liposomes and erythrocytes serve as well-established models for studying the radical processes in biological membranes. In addition to their simplicity and high susceptibility to oxidative damage,^23–25^ erythrocyte membranes share key biophysical features with neuronal membranes, including similar lipid compositions and organized membrane protein asymmetry, as evidenced by comparative lipidomic analyses of erythrocyte and neuronal plasma membranes.^26–28^ Erythrocytes, due to their oxygen transport function and rich polyunsaturated lipid membrane content,^29,30^ provide an excellent model for examining the antioxidant effects of ibogalogs, as they are highly susceptible to oxidative stress and suitable for preliminary screening prior to application in more complex neuronal systems.

Despite increasing interest in the behavioral and serotonergic effects of ibogalogues, their antioxidant potential and interactions with biological membranes have not been systematically explored. To address this gap, we investigated the redox activity of three structurally related compounds: ibogaminalog (DM506), tabernanthalog (TBG), and ibogainalog (IBG), using complementary in vitro and ex vivo membrane models, including liposomes, erythrocytes, and rat synaptosomes, under conditions of 2,2′-azobis­(2-amidinopropane) dihydrochloride (AAPH)-induced oxidative stress (see Figure 1).

**Figure 1:**
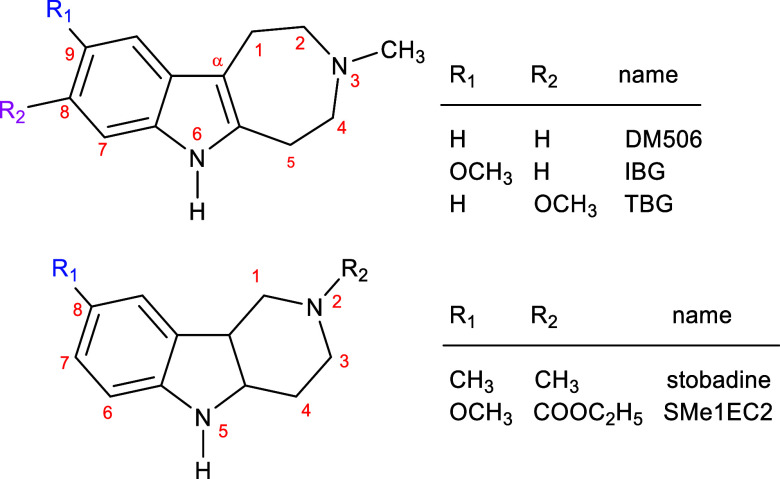
Molecular structure of neutral (base) forms of ibogalogs, including DM506 (ibogaminalog), IBG (ibogainalog), and TBG (tabernanthalog), as well as structurally similar antioxidants such as stobadine and its derivative SMe1EC2. Please note that the atom numbering differs between the two structures. Therefore, for the purpose of this discussion, we also use the names of the substituent positions *meta* (magenta) and *para* (blue) (with respect to the N atom in the aromatic ring).

To clarify the physicochemical mechanisms by which ibogalogs might protect lipid membranes from peroxidation induced by AAPH and consequently reduce oxidative stress, we assessed their antioxidant and membrane-stabilizing effects in both synaptosomal preparations and simplified membrane models. The use of controlled membrane model systems enables structure–activity relationship analysis and mechanistic dissection of radical-induced membrane damage without confounding intracellular signaling pathways. The inclusion of synaptosomes provides a biologically relevant neuronal membrane system, bridging simplified models and neurochemical contexts. Our results suggest that ibogalogs may exert protective effects through a direct antioxidant mechanism that limits lipid peroxidation complementing their established neuromodulatory actions on 5-HT receptors.^6–10^ This dual mode of action is further supported by theoretical modeling, which links antioxidant potency to the position of methoxy groups in the indole ring as a determinant of radical stability and redox behavior. Taken together, our study highlights the physicochemical properties of ibogalogs underlying their redox activity and hydrogen atom transfer characteristics, providing a mechanistic framework relevant to oxidative stress-associated neurobiological contexts, such as epilepsy and neurodegenerative disorders.^31–33^

## 2 Results and Discussion

Previous findings from various biological models have indicated that the alkaloid ibogamine exhibits antioxidant, cytoprotective, antiaddictive, and potential neuroprotective properties.^6,8,11,13,18^ Ibogamine is the parental molecule of ibogaminalog (DM506), which preserves the azepinoindole rings found in iboga alkaloids.^10^ This structural resemblance opens the door for the antioxidant activity of ibogalogs. However, the exact mechanism of their action remains unclear. Thus, we evaluated the potential antioxidant and membrane-protecting activity of DM506, TBG, and IBG in two biologically relevant systems: erythrocytes and synaptosomes, and we confronted the results with the kinetic measurements in model membranes. Finally, to explain our experimental findings, we calculated using quantum chemical methods thermodynamic parameters, namely, C–H and N–H bond strengths (the bond dissociation enthalpies) and ionization potentials, for all three ibogalogs and compared them with two known antioxidant/neuroprotective compounds, namely, stobadine and its derivative SMe1EC2 (Figure 1).^34–37^

### 2.1 Ibogalogs Prevent Erythrocyte Hemolysis

To investigate the protective effects of ibogalogs against oxidative damage in biological membranes, human erythrocytes were used as a model system. Their unique structural characteristics, e.g., lacking a nucleus and organelles, facilitate the interpretation of membrane-level processes. Erythrocytes are widely recognized as suitable models for studying lipid peroxidation and oxidative stress-related damage to membrane integrity.^23,29,38,39^ As a first step, we evaluated whether ibogalogs induce any structural damage to erythrocytes by assessing hemolysis across a wide concentration range (0.01 to 10 μM). No hemolytic activity was observed for DM506, IBG, or TBG at any tested concentration (one-way ANOVA followed by Tukey’s posthoc test; Figure 2A). Statistical analysis confirmed the absence of meaningful membrane disruption, as effect sizes (Cohen’s *d*) for hemolysis relative to control remained small across all concentrations for all tested compounds (specifically *d* < 0.3 for TBG; maximum *d* = 0.31 at 0.1 μM). Confidence intervals (CIs) for mean hemolysis values overlapped with those of the control group, further supporting the lack of a significant effect. For example, at 10 μM, the 95% CI for TBG was 1.49–2.09 vs 1.17–2.57 for control. Similar trends were observed for IBG and DM506, with no dose-dependent increase in hemolysis or deviation from baseline. These results indicate that DM506, IBG, and TBG do not compromise erythrocyte membrane integrity, thereby indicating that the indole-hexahydroazepine rings found in ibogalogs do not induce cytotoxic effects, supporting previous results with the whole structure of iboga derivatives.^40,41^

**Figure 2:**
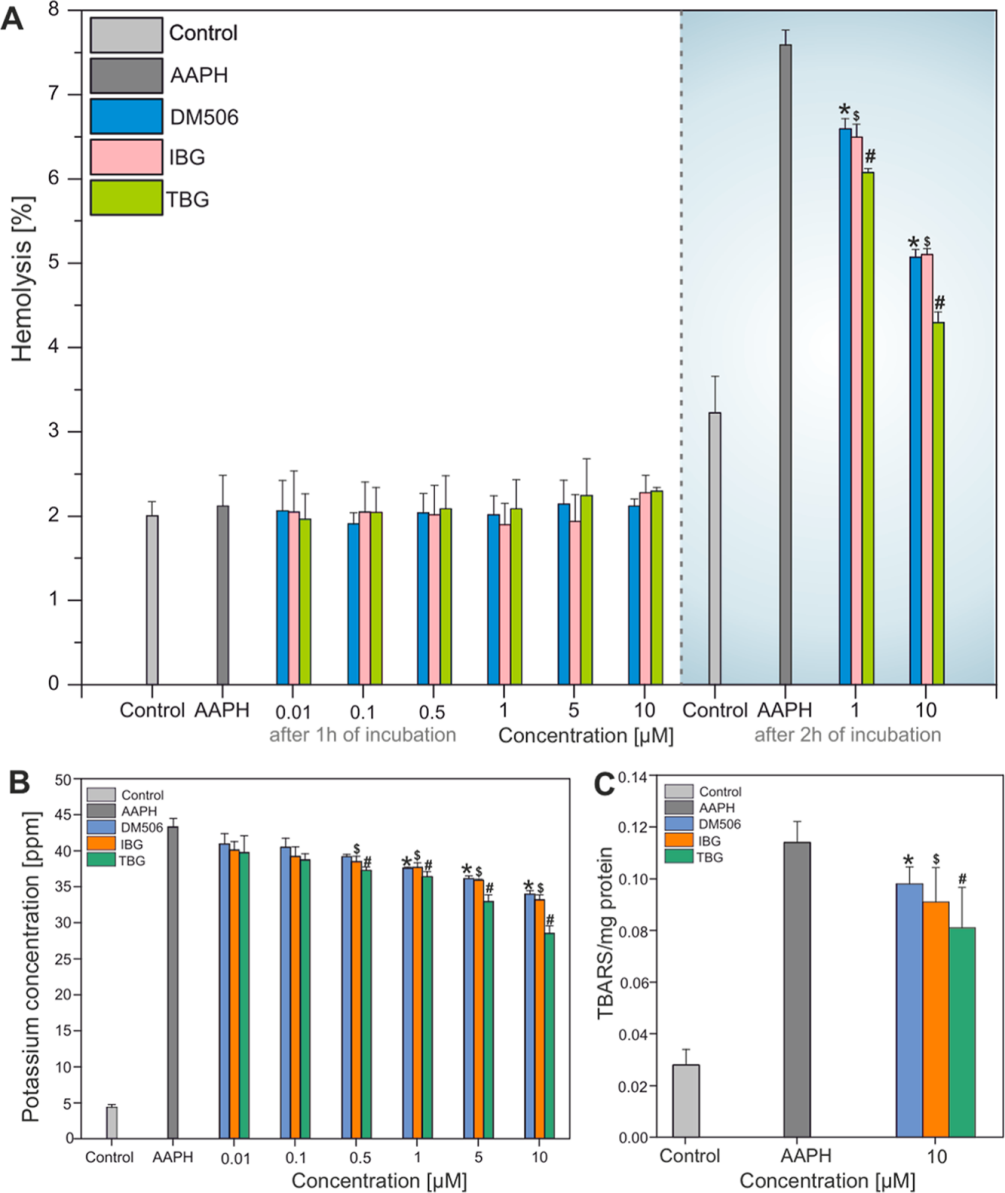
Protective effects of ibogalogs against 50 mM AAPH-induced oxidative damage in human erythrocytes. (A) After 2 h of incubation, 50 mM AAPH triggered significant hemolysis, which was effectively prevented by ibogalogs. (B) AAPH induced K^+^ leakage after 1 h, indicative of early membrane damage, while ibogalogs markedly reduced potassium efflux. (C) Lipid peroxidation, assessed by malondialdehyde (MDA) formation after 1 h of incubation with AAPH, was significantly reduced in the presence of ibogalogs, indicating their efficacy in attenuating oxidative membrane damage. (*; DM506); ($; IBG); (#; TBG), vs AAPH conditions, one-way ANOVA followed by Tukey’s posthoc test (*n* = 3). The lipophilic nature of ibogalogs may facilitate their membrane penetration and enhance their efficiency in scavenging radicals. However, the ability of ibogalogs to lower TBARS levels (see Figure 2C) reinforce the notion that their antioxidant activity extends beyond simple radical scavenging. The lipophilic properties likely facilitate the interaction of ibogalogs with the hydrophobic core of the lipid bilayer, enhancing their efficacy in intercepting lipid radicals at the membrane level.

After 1 h of incubation, 50 mM AAPH did not induce hemolysis, and the ibogalogs themselves did not trigger adverse effects. However, extending the incubation to 2 h resulted in substantial AAPH-induced hemolysis (7.59 ± 0.13; 95% CI = 7.02–8.16), which was effectively attenuated by ibogalogs. At 1 μM, DM506, IBG, and TBG reduced hemolysis by 13.3%, 14.5%, and 19.9%, respectively, compared to the AAPH group (mean hemolysis values: 6.59 ± 0.09 for DM506, 6.49 ± 0.12 for IBG, and 6.08 ± 0.04 for TBG). At 10 μM, the reductions were even more pronounced: 33.2% for DM506 (5.07 ± 0.07), 32.7% for IBG (5.11 ± 0.05), and 43.4% for TBG (4.30 ± 0.09). These effects were statistically significant (*p* < 0.01 or *p* < 0.001 vs AAPH; one-way ANOVA followed by Tukey’s posthoc test), with corresponding effect sizes (Cohen’s *d*) ranging from −5.07 (for IBG 1 μM) to −16.57 (for TBG 10 μM). Confidence intervals for the treated groups did not overlap with those treated with AAPH, further supporting the strength of the protective effect (specifically for TBG at 10 μM 95% CI = 3.90–4.70). Collectively, these results indicated that ibogalogs effectively counteract free-radical-induced membrane damage in erythrocytes in a concentration-dependent manner (Figure 2A). Our findings support the findings of limited previous studies on the protective effects of well-characterized iboga derivatives on antioxidant enzymes in erythrocytes^18^ and on the overall antioxidant properties.^42^

### 2.2 Ibogalogs Prevent Potassium Leakage in Erythrocytes

We also assessed the effect of the tested compounds (DM506, IBG, or TBG) on potassium leakage from erythrocytes in the absence of AAPH. No significant changes in K^+^ efflux was observed following 1 h incubation with any of the ibogalogs tested at a 0.01–10 μM concentration range, indicating that these compounds do not disrupt membrane ion permeability under basal conditions (one-way ANOVA followed by Tukey’s posthoc test, Figure 2B). This conclusion is supported by small to moderate effect sizes (Cohen’s d), all ranging from −0.21 to −1.15 when compared to control. Furthermore, 95% CI for all concentrations overlapped with the control range (specifically for TBG at 10 μM 95% CI = 3.49–5.31 vs control 3.49–5.31), reinforcing the absence of a biologically relevant effect on potassium permeability. Further studies examined the effects of ibogalogs under oxidative stress induced by 50 mM AAPH. Although AAPH did not induce hemolysis after 1 h of incubation as indicated in our earlier studies,^23^ it caused a marked increase in K^+^ leakage due to early oxidative alterations in the erythrocyte membrane (Figure 2B). This was confirmed by a dramatic increase in potassium efflux from 4.39 ± 0.21 in the control group to 43.31 ± 1.29 in the AAPH-treated group (95% CI = 37.76–48.86). The corresponding effect size was extremely large (Cohen’s *d* = 24.31), indicating significant membrane damage. Potassium efflux is considered a more sensitive and early biomarker of membrane damage, preceding hemolysis and reflecting oxidative stress-induced impairment of membrane proteins.^23^ Subsequently, we evaluated whether preincubation with each ibogalog could modulate the AAPH-induced K^+^ leakage. TBG and IBG applied at concentrations ranging from 0.5 to 10 μM significantly reduced potassium efflux compared to cells treated with AAPH alone. DM506 reduced potassium efflux by 13.2%, 16.5%, and 21.6% at concentrations of 1, 5, and 10 μM, respectively (*p* < 0.001 vs AAPH-treated group, one-way ANOVA followed by Tukey’s posthoc test). IBG caused a reduction by 8.8% (*p* < 0.05), 12.9%, 17.1%, and 23.4% at 0.5, 1, 5, and 10 μM, respectively (*p* < 0.001 for 1 – 10 μM). TBG reduced potassium efflux by 13.9% (*p* < 0.01), 16.0%, 23.9%, and 34.1% at 0.5, 1, 5, and 10 μM, respectively (*p* < 0.001 for 1–10 μM). These effects were supported by large effect sizes relative to the AAPH group, with Cohen’s *d* values ranging from −2.1 to −5.8 across compounds and concentrations tested. In addition, 95% CI for K^+^ efflux at all effective concentrations did not overlap with the AAPH group (specifically for TBG at 10 μM: 95% CI = 25.98–31.07 vs AAPH 95% CI = 37.76–48.86), confirming a significant reduction of oxidative membrane damage. These results suggest a protective role of all tested ibogalogs, with TBG being most effective against early oxidative membrane damage (Figure 2B). Considering that 10 μM TBG does not affect Na^+^/K^+^-ATPase function,^10^ it is unlikely that ibogalogs exert a protective effect by directly maintaining the K^+^ gradient in erythrocytes, as reported for some nutritional antioxidants.^43^ Protection against K^+^ leakage is most likely due to antioxidant action and membrane stabilization, rather than to a direct effect on the Na^+^/K^+^ pump. The protective effect may result from both the scavenging of radicals generated by AAPH and membrane stabilization through interactions with the lipid bilayer.

### 2.3 Ibogalogs Reduce Thiobarbituric Acid-Reactive Substances Levels in AAPH-Treated Erythrocyte Ghosts

Isolated erythrocyte membranes were used as a model system to assess lipid peroxidation levels by measuring thiobarbituric acid-reactive substances (TBARS), according to the protocol described in reference 23. Membrane samples were incubated for 1 h with 50 mM AAPH to induce oxidative stress in the absence or presence of 10 μM of each ibogalog (Figure 2C). All three ibogalogs used at 10 μM significantly reduced the levels of TBARS compared to the AAPH-treated group. Specifically, DM506, IBG, and TBG decreased TBARS levels by 17.5%, 20.2%, and 25.4%, respectively. This reduction in lipid peroxidation was statistically significant (*p* < 0.05, one-way ANOVA followed by Tukey’s posthoc test), demonstrating the protective effect of ibogalogs against AAPH-induced oxidative damage in erythrocyte membranes. These effects were supported by medium-to-large effect sizes relative to the AAPH group, with Cohen’s *d* values ranging from −1.21 to −1.82 across ibogalogs at 10 μM. In addition, 95% confidence intervals for TBARS levels at all tested ibogalogs did not significantly overlap with the AAPH group (specifically for TBG at 10 μM Cohen’s *d* = – 1.82, 95% CI = −4.52 to 0.88), confirming a biologically meaningful reduction of oxidative membrane damage. Our results are consistent with previous studies, demonstrating that crude extracts of *Tabernaemontana catharinensis*, rich in ibogamine, exhibit significant in vitro antioxidant properties, as evidenced by the inhibition of TBARS production and DPPH radical scavenging activity.^44^ However, there is limited but noteworthy evidence suggesting that ibogamine may also exert pro-oxidative effects. Specifically, the scarce literature data indicate that the pro- or antioxidative activity of ibogamine treatment including TBARS levels in liver, kidney, and heart tissues is both concentration-dependent and modulated by the time elapsed after administration as well as the sex of the animals.^42,45^

### 2.4 Ibogalogs Reduce Thiobarbituric Acid-Reactive Substances Levels in Isolated Synaptosomal Fractions

Building upon the findings obtained from model biological membranes, we proceeded to investigate the antioxidant properties of ibogalogs in a more complex system representative of neuronal tissue. To this end, we employed synaptosomes isolated from the hippocampus (HPC) and frontal cortex (FC), two brain regions highly susceptible to oxidative damage^46,47^ and critically involved in memory processing and cognitive function.^48,49^ They were used as an ex vivo model to assess whether the membrane-stabilizing and antioxidative effects observed in erythrocytes would also be apparent in neuronal tissue under conditions of oxidative stress (Figure 3). Samples were incubated with 50 mM AAPH for 1 h in the absence or presence of 10 μM ibogalog (Figure 3). Treatment with ibogalogs significantly reduced the TBARS levels compared to AAPH-treated treatment (10 μM DM506, IBG, and TBG caused a reduction in TBARS levels in HPC by 16.0%, 18.4%, 28.4%, respectively, and in FC by 16.0%, 19.6%, 19.9%, respectively vs AAPH conditions). This reduction in lipid peroxidation in synaptosomal membranes derived from both HPC and FC tissues was statistically significant (*p* < 0.05, one-way ANOVA followed by Tukey’s posthoc test), confirming the protective effect of ibogalogs against oxidative damage of hippocampal and cortical synaptosomal membranes. These effects were further supported by quantitative effect size analysis. In hippocampal synaptosomal membranes, all three ibogalogs reduced TBARS levels compared to the AAPH-treated group, with Cohen’s *d* values of −0.78 (DM506), −0.83 (TBG), and −1.52 (TBG). In FC, similar reductions were observed, with Cohen’s *d* values of −0.87 (DM506), −0.91 (IBG), and −0.91 (TBG). Among these, TBG in HPC showed the strongest effect, with a Cohen’s *d* of −1.52 and a 95% confidence interval of −3.49 to 0.44.

**Figure 3:**
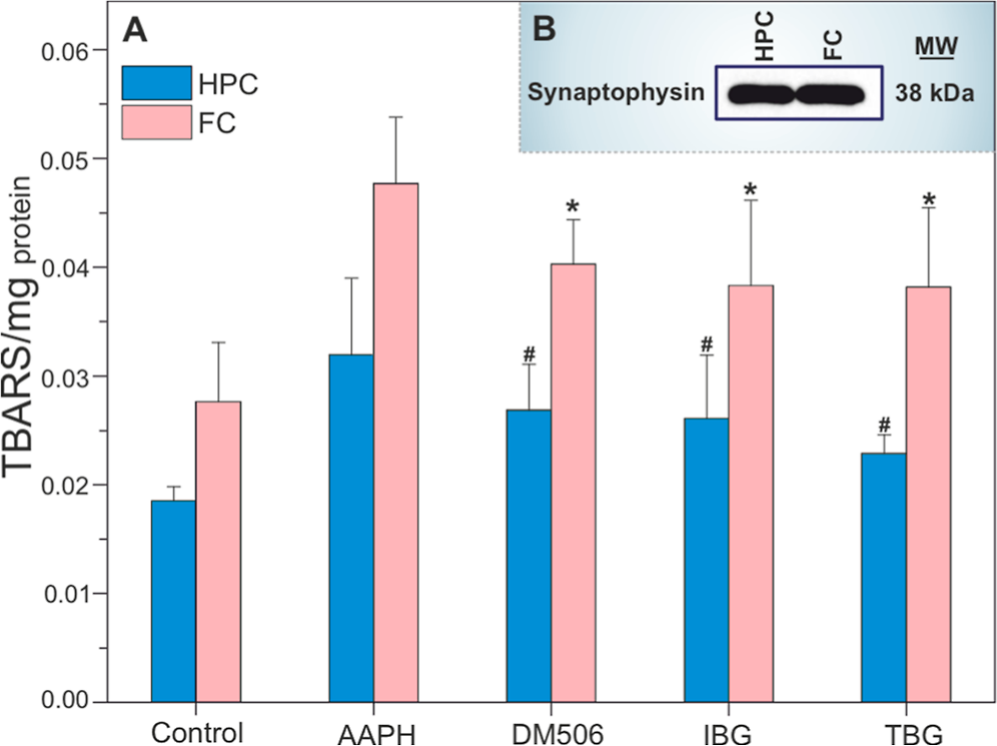
(A) Ibogalogs reduce the TBARS level in hippocampal (HPC) and frontal cortex (FC) synaptosomal fractions compared to AAPH-treatment, one-way ANOVA followed by Tukey’s posthoc test (*n* = 4). (B) Western blot verification of synaptophysin levels (synaptosomal marker) in synaptosomes prepared from rat HPC and FC. The molecular weight of the loaded protein (5 μg/lane) was verified with PageRuler Plus Prestained Protein Ladder.

These results confirm that ibogalogs not only preserve the structural integrity of erythrocyte membranes but also exert a comparable protective effect on neuronal synaptic membranes, which are particularly vulnerable to oxidative damage due to their high content of polyunsaturated fatty acids and metabolic activity.^50,51^ The observed decrease in TBARS levels suggests that ibogalogs effectively counteract lipid peroxidation processes initiated by free radicals in the HPC and FC synaptic membranes. This reinforces the hypothesis that their membrane-protective properties are not only limited to peripheral cells but also applicable to nervous system components. Unexpectedly, TBARS levels were higher in synaptosomal fractions obtained from FC than HPC. This regional difference may reflect variations in synaptic activity, lipid composition, and antioxidant capacity. Despite HPC being rich in glutamatergic transmission and PUFA content, FC synaptic membranes might be more susceptible to lipid peroxidation under basal conditions.^52^

Despite some confidence intervals including zero, consistently negative Cohen’s *d* values across hemolysis, potassium leakage, and TBARS levels suggest a biologically relevant protective effect of ibogalogs on membrane integrity. These effects were particularly pronounced for TBG, which showed the strongest reduction in the extent of oxidative damage. Taken together with ANOVA-based significance across both erythrocyte and neuronal synaptosome models, these findings highlight the consistent antioxidant and membrane-stabilizing action of ibogalogs under oxidative stress.

### 2.5 Antioxidant Activity of Ibogalogs in Liposomal Systems

Figure 4 presents the kinetic profiles of oxygen consumption during the oxidation of methyl linoleate, initiated by 10 mM AAPH, in a liposomal system at pH 7.4, containing either ibogalog or PMHC. PMHC, the most active natural antioxidant belonging to the class of radical trapping antioxidants, was used as a standard phenolic antioxidant. Both PMHC and α-tocopherol share a similar structure, featuring a 6-hydroxychroman ring. The key difference between them is that PMHC lacks the phytyl chain, a component that does not directly contribute to radical scavenging activity but slightly affects the mobility of the antioxidant in lipid membranes.

**Figure 4:**
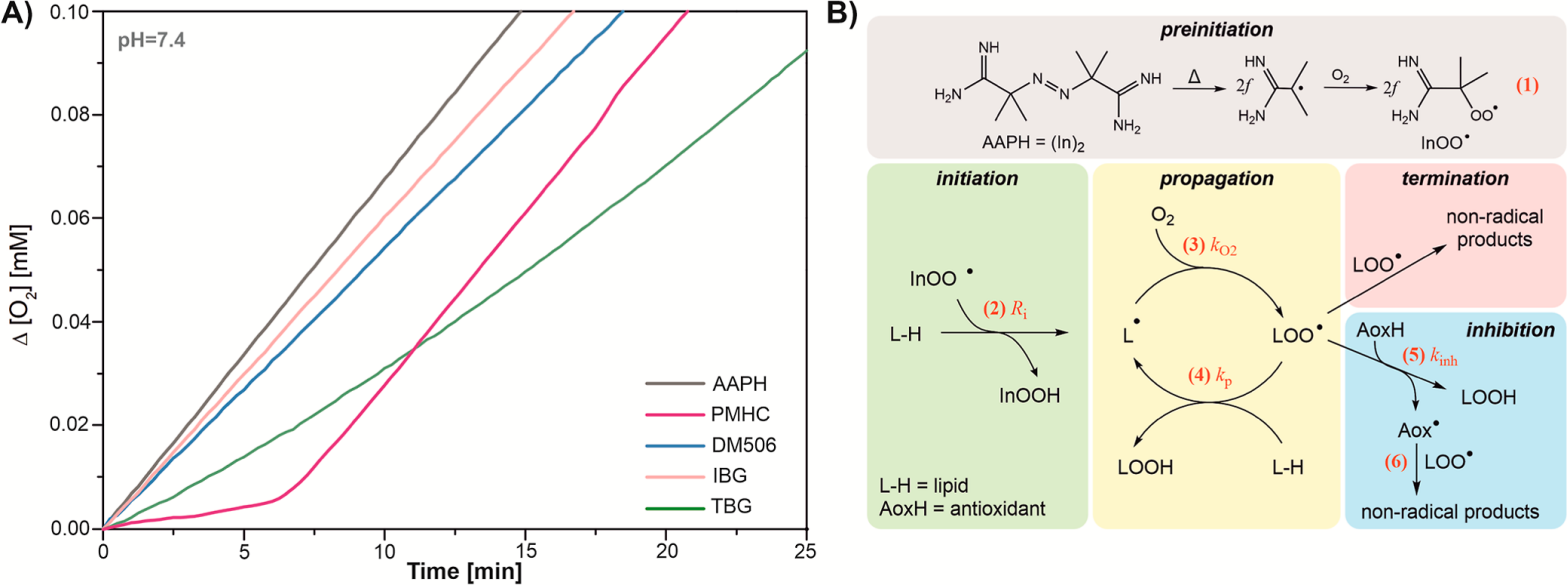
(A) Oxygen consumption curves for the autoxidation of DMPC containing methyl linoleate initiated by 10 mM AAPH at pH 7.4 and 37 °C, in the presence of 10 μM DM506, IBG, and TBG, respectively, or 1 μM PMHC (used as a reference antioxidant). (B) Thermal decomposition of the AAPH initiator and formation of peroxyl radicals during preinitiation (process 1). The initiator decomposes into two radicals with yield *f*, meaning 1 mole of (In)_2_ produces *2f* radicals capable of attacking lipids. Lower panels: three stages of lipid autoxidation (peroxidation) with kinetically important reactions (2–6). *R*_*i*_ denotes the rate of initiation (reaction 2), while *k*_O2_, *k*_p_, and *k*_inh_ are the rate constants for reactions 3, 4, and 5, respectively. See the text for details.

Peroxidation is a chain process mediated by lipid peroxyl radicals, LOO^•^. The mechanism of this process is presented in Figure 4B, together with reactions 1–6. Key kinetic parameters for this process can be obtained when the rate of initiation is controlled using an azo-initiator (here, AAPH, reactions 1 and 2 in Figure 4B). These primary peroxyl radicals then initiate the peroxidation chain (propagation), with reaction 3 occurring rapidly and reaction 4 being the rate-determining step. For lipids containing linoleate derivatives, such as linoleic acid, methyl linoleate, and phospholipids containing the linoleic acyl group, the propagation rate constant, *k*_p_, is within the range of 15–100 M^–1^s^–1^ (see data collected in Supporting Information to our previous article^25^). The process will be inhibited in the presence of of antioxidants (AoxH) responsible for trapping peroxyl radicals (reactions 5 and 6 in Figure 4B). For active antioxidants (*k*_inh_ ≫ 10^2^ *k*_p_), even micromolar concentrations are sufficient to suppress peroxidation, which is manifested as an induction period. After complete consumption of the antioxidant, the peroxidation accelerates. Indeed, when PMHC is added to the liposomal system, a distinct induction period of ∼7 min can be observed (Figure 4A), and the rate of initiation, *R*_*i*_, can be easily determined from the following equation:

1 $$R_{i}=n{\left[AoxH\right]}_{0}/\tau$$

where [AoxH]_0_ is the starting concentration, and *n* is the number of radicals reduced per one molecule of an antioxidant. For PMHC, *n* = 2.0. For the studied compounds, the parameter *n* is not known, but when their activity is sufficient to form the induction period, the parameter *k*_inh_ can be calculated from a linear function of oxygen uptake, denoted as [O_2_]_*t*,_ plotted versus ln­(1–*t*/τ), see eq 2. A detailed description related to the calculation of kinetic parameters can be found in a previous study.^53^

2 $${-\Delta \left[O_{2}\right]}_{t}=\frac{k_{p}\cdot \left[LH\right]}{k_{inh}}\cdot \ln\left(1-\frac{t}{\tau }\right)$$

The parameters τ, *R*_inh_, and *R*_ox2_ are listed in Table 1. The τ values obtained for PMHC were used to calculate the initiation rate, yielding *R*_*i*_ = (4.9 ± 0.3) nMs^–1^ at pH 7.4. In the presence of 1 μM PMHC, the reaction follows a chain mechanism even during the inhibition period (ν_inh_ > 3), and the *k*_inh_ calculated for this antioxidant is consistent with literature values.^25^ In the presence of DM506 or IBG added to liposomes, we observed slower peroxidation, however, without an induction period, and such kinetic behavior is called retardation. On the other hand, the curve for TBG exhibited an intermediate pattern: There is a deviation from the straight line and a very slight inflection between the third and ninth minute after the injection. The kinetic profile begins to resemble the curve typical of a sample containing an antioxidant, but inhibition is minimal (ν_inh_ = 9.5 vs ν_ox2_ = 16), and the induction period is not distinctly visible. These observations suggest that ibogalogs inhibit lipid peroxidation in liposomal systems at pH 7.4, with one of them (TBG) exhibiting antioxidant properties. The parameter *n* = 0.3, calculated for TBG (10 μM, τ = 9.5 min) using transformed eq 1, suggests a moderate capability to trap peroxyl radicals, perhaps due to back reactions or formation of a stable adduct that does not further react with another peroxyl. This ibogalog acts as a relatively weak antioxidant with a *k*_inh_ value of 7000 ± 300 M^–1^ s^–1^. IBG and DM506 do not exhibit an induction period, but they cause a retardation effect, suggesting that *k*_inh_ is well below 10^3^ M^–1^ s^–1^. Thus, regardless the mechanism, the rate of reaction 5 is not competitive with the rate of propagation and an induction period is not manifested. Such kinetic behavior does not exclude DM506, IBG, and TBG as good antioxidants or redox mediators in other systems where oxidative stress is caused by ROS other than peroxyls.

**Table 1:** Parameters Determined for Initiated Autoxidation of DPMC Liposomes Containing Methyl Linoleate in the Presence of 1.0 μM PMHC or 10 μM Ibogalog at pH 7.4^a^ ^,^ ^b^ ^,^ ^c^

Parameter	Compound				
	none	PMHC	DM506	IBG	TBG
*R*_ox1_ [nM s^–1^]	111 ± 8	107 ± 8	111 ± 13	135 ± 4	144 ± 10
ν_ox1_ ^c^	23 ± 2	22 ± 2	23 ± 3	28 ± 1	30 ± 2
τ [min]	-	6.9 ± 0.5	-	-	9.5 ± 0.9
*R*_*i*_ [nM s^–1^]^e^	4.9 ± 0.3	4.9 ± 0.3	4.9 ± 0.3	4.9 ± 0.3	4.9 ± 0.3
*R*_inh_ [nM s^–1^]	-	22 ± 0.4	98 ± 11^d^	102 ± 7^d^	46 ± 5
*R*_ox2_ [nM s^–1^]	-	105 ± 10	100 ± 10^d^	102 ± 5^d^	77 ± 5
ν_*i*nh_ ^b^	-	4.6 ± 0.8	20 ± 2.2^d^	21 ± 1.4^d^	9.5 ± 1.0
ν_ox2_ ^c^	-	22 ± 2	21 ± 2	21 ± 1	16 ± 1
*k*_inh_ × 10^–4^ [M^–1^ s^–1^]^f^	-	4.6 ± 0.3	-	-	0.7 ± 0.03

a Induction period length (τ), autoxidation initiation rate (*R*_*i*_), inhibited autoxidation rate (*R*_inh_), inhibition rate constants (*k*_inh_), rates of non-inhibited and post-inhibition processes (*R*_ox1_ and *R*_ox2_, respectively), and the kinetic chain length of peroxidation (ν_ox1_, ν_*i*nh_; ν_ox2_).

b ν_*i*nh_ = *R*_inh_/*R*_*i*_.

c ν_ox1_ = *R*_ox1_/*R*_*i*_ and ν_ox2_ = *R*_ox2_/*R*_*i*_.

d ν_*i*nh_ and *R*_inh_ calculated for the retarded process.

e *R*_*i*_ for all systems calculated with PMHC from eq 1.

f Parameter *k*_inh_ was calculated from eq 2, with *k*_p_ = 41 M^–1^s^–1^ for liposomes (see ref 25).

The different positions of the methoxy group in IBG and TBG, and the lack of a methoxy group in DM506, seem to be an important factor for the differences in antioxidant activity among ibogalogs. The presence of a methoxy group in IBG resembles the position in the well-known antioxidant SMe1EC2 (Figure 1).^34,35,54,55^ It seems that the methoxy functionality greatly enhances the bioactivity of SMe1EC2 as it protects against oxidative damage, including in cellular systems and in models using the Fe/ascorbic acid system, effectively protecting lipids and creatine kinase.^35^ Additionally, SMe1EC2 demonstrated beneficial neuroprotective effects, both in rats and microglial cells.^54^ However, TBG demonstrated greater efficacy as a protective and antioxidant agent than IBG under the tested conditions. This surprising observation encouraged us to take a closer look at the structural differences of DM506, IBG, and TBG compared with the two well-known compounds: stobadine and SMe1EC2.

### 2.6 Quantum Chemical Calculations

The bond dissociation enthalpy (BDE) values for the N–H and C–H bonds of the three studied compounds, as well as for stobadine and its derivative SMe1EC2, are summarized in Table 2. We employed a newly developed density functional theory approach where the results produced by the ωB97-D/pcseg-1 are significantly enhanced with the use of optimized atom-centered potentials (ACPs), see below. To validate the computational approach, gas-phase BDEs for stobadine were calculated by using the (RO)­CBS-QB3 method, which is known to reproduce experimental BDE values with high accuracy. The comparison shows a mean absolute error of 0.64 kcal/mol (mean signed error −0.43 kcal/mol) across seven bonds, with the largest deviation (2.3 kcal/mol) observed for bond “e” (see Table 2). Analogous calculations were also performed using M06-2X/def2-TZVPP: this level of theory produced mean absolute (signed) errors relative to (RO)­CBS-QB3 of 1.15 (0.56) kcal/mol. Taken together with the results obtained for aniline and the indole series, these findings support the reliability of the ACP-based computational approach used in this study. The antioxidant, free radical scavenging, and neuroprotective activity of stobadine and SMe1EC2 are described in the literature.^34–37^ It is important to note that in these derivatives the indole moiety is reduced to an indoline structure (2,3-dihydro-1*H*-indole), whereas in the three ibogalogs examined here, the aromatic indole framework remains intact. This structural difference, although subtle, leads to a markedly different ordering of C–H and N–H bond strengths. In stobadine and SMe1EC2, the weakest bond, according to the calculations, is the N–H bond (Table 2), in the gas phase, in nonpolar solvents, and in water. For SMe1EC2, the difference between the weakest bond (N–H) and the next weakest bond (the C–H bond labeled “f”) is 5 kcal/mol. In stobadine, the difference between the BDE for N–H and C–H is less pronounced (1.1 and 2.5 kcal/mol in the gas phase and in water, respectively). However, compounds containing the indole moiety (instead of indoline) exhibit a reverse order in the strengths of the bond, with the C–H bond being weaker than the N–H bond. The only exception is TBG in water, where BDE (N–H) = 85.5 kcal/mol followed by C–H at position “b” (86.3 kcal/mol).

**Table 2:**
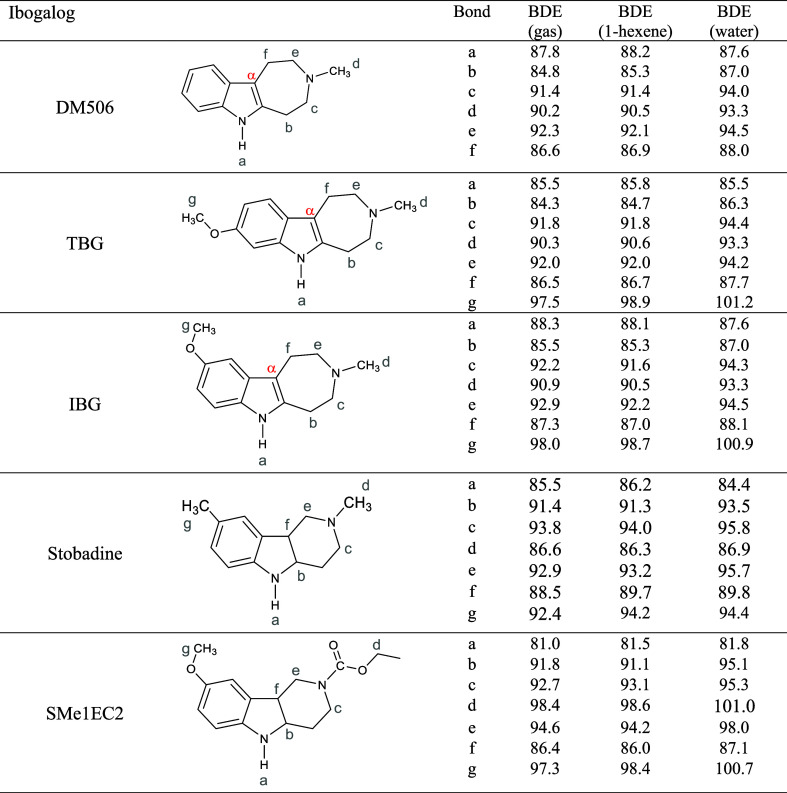
C–H and N–H Bond Dissociation Enthalpies, in kcal/mol, Computed in the Gas Phase, 1-Hexene Solvent, and Water Solvent for Ibogalog Systems

Although the calculated differences in bond strengths do not rule out hydrogen abstraction (hydrogen atom transfer, HAT) from either N–H or C–H sites, the N–H bond HAT from heteroatoms can be faster than that from C–H bonds, even when the N–H bond is thermodynamically stronger. For example, in 9,10-dihydroacridine (DHAC), the calculated BDE­(C–H) is approximately 71.0 kcal/mol, while the BDE (N–H) is about 82.3 kcal/mol. Despite this, hydrogen atom transfer from the N–H group is strongly favored over transfer from the C–H bond.^56^ A similar phenomenon was reported by Barclay et al.^57^ for dipyrrinones (bilirubin model compounds), where the hydrogen atom is abstracted from the heteroatom, despite the N–H bond being significantly stronger than the C–H bond (84.7 kcal/mol vs 70.1 kcal/mol). The preferential HAT from stronger N–H bonds in many heterocyclic compounds is a kinetic phenomenon driven by the efficiency of the reaction mechanism (proton coupled electron transfer or single electron transfer (SET)), which is also significantly influenced by the polarity of the solvent and the nature of the attacking radical. These factors combine to make the reaction kinetically more favorable for the N–H bond, overriding the thermodynamic advantage of a weaker C–H bond. On the other hand, C–H bonds in proximity to heteroatoms often react more readily than would be predicted based solely on their BDEs, making steric and stereoelectronic effects (for example, secondary orbital interactions) as important as the overall thermodynamics.^58^

While a detailed determination of whether hydrogen donation to the peroxyl radical occurs from a C–H or N–H bond is beyond the scope of this work, we nevertheless attempted to clarify the role of the indole moiety in the radical-reduction mechanism of the DM506, IBG, and TBG series. We calculated the N–H BDEs in a series of indoles (Figure 5). The results reveal that substituting dimethylindole with a methoxy group at the *para* and *meta* positions relative to the nitrogen atom yields somewhat unexpected outcomes: although both substituents lower the N–H BDE, *meta* substitution leads to a lower N–H BDE than *para* substitution.

**Figure 5:**
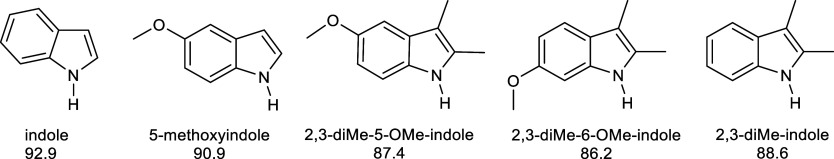
Calculated gas-phase N–H BDEs (in kcal/mol) for a series of anilines and indoles using the atom-centered potential (ACP) approach.

However, the gas-phase N–H BDEs calculated for the ibogalogs display a counterintuitive trend, with TBG exhibiting the lowest value (85.5 kcal/mol), followed by DM506 (87.8 kcal/mol) and IBG (88.3 kcal/mol). To further investigate the surprising effect of methoxy functionality, the gas-phase spin distributions at the ωB97XD/pcseg-1-ACP level of theory for the radicals associated with DM506, TBG, and IBG were calculated (see Table S2 and Figure S1). Analysis of the calculated spin-density distributions (Supporting Information) shows that, following HAT from the indole nitrogen, the unpaired electron is predominantly localized on the five-membered ring, at the carbon atom labeled α (see Figure 1 and Table 2). Therefore, the stability of the resulting radical is enhanced by the methoxy group at position *para* relative to the α-carbon (i.e., *meta* relative to the nitrogen atom), making the radical formed from TBG more stable than the radical formed from IBG. It is worth noting that the above trend applies only to the family of compounds containing an indole ring. In contrast, stobadine and SMe1EC2 contain an indoline moiety (i.e., without a double bond in the indole system). As a result, the nitrogen atom, not the α-carbon, holds the majority of the unpaired spin density. In both stobadine and SMe1EC2, the absence of the double bond reveals the expected substituent effect associated with placing a methoxy group in the *para* position relative to the nitrogen. However, the magnitude of this effect is quite pronounced (cf. 4.1 kcal/mol vs 3.4 kcal/mol in aniline). This is likely due to a change in the planarity at the nitrogen (N = H) center upon substitution. In stobadine, the sum of the three angles at the nitrogen is 336.7°, while in SMe1EC2, it increases to 348.5°. The greater planarity in the latter allows for better conjugation of the nitrogen lone pair with the aromatic ring in the parent compound, leading to destabilization through repulsive electron-donating interactions of two functionalities (N and OCH_3_) into the aryl system.

Interestingly, rate constants for H atom abstraction by the hydroxyl radical from melatonin, calculated using quantum chemical methods by Galano and Reiter,^59^ indicated that the process can occur from multiple sites with significant probability. Abstraction from the N–H group of the indole moiety comprised only 10.1% of the overall HAT rate constant, and this number decreased to 4% in aqueous solution. HAT from several other C–H sites contributes to the remainder of the overall rate constant. For abstraction by the hydroperoxyl radical, only one C–H site (the equivalent to site “e” in SMe1EC2—see Table 2) was found to be relevant for HAT.

We also evaluated the vertical ionization potential of all five compounds to determine reactivity via mechanisms involving electron transfer as the initial step, specifically, SET or sequential electron transfer–proton transfer (SETPT). Ionization potential values (Table 3) indicated that TBG is more prone to forming a radical cation than the other compounds, such as SMe1EC2.

**Table 3:** Vertical Ionization Potentials, in kcal/mol, Calculated in the Gas Phase, 1-Hexene, and Water Solvent for Ibogalog Systems

Ionization Potential			
Ibogalog	(gas)	(1-hexene)	(water)
DM506	170.4	146.9	127.8
IBG	169.5	146.5	128.0
TBG	164.4	141.9	124.6
Stobadine	176.0	150.7	130.8
SMe1EC2	168.8	144.5	126.1

Although this result was not surprising, as discussed above, SET and SETPT mechanisms are typically favored for strong oxidizing agents such as hydroxyl or trichloromethylperoxyl radicals. In contrast, mechanistic studies of alkylperoxyl radicals reacting with melatonin^59^ suggest rather limited contribution of SET-related mechanisms to the overall kinetics.

In summary, theoretical calculations show that N–H and C–H bond reactivity is strongly governed by the indole/indoline framework, substituent pattern, and electronic effects. Integration of kinetic lipid peroxidation data with BDE calculations reveals that the position of the methoxy substituent critically modulates the radical stability and antioxidant performance. This mechanistic link explains the superior activity of TBG compared with that of IBG and DM506.

### 2.7 Interaction with Lipid Membranes

The different abilities of ibogalogs to inhibit peroxidation in liposomal systems might be a consequence of at least two features, their reducing power and ability to interact with lipid membranes. Since the experiments were conducted using electrically neutral DMPC liposomes, the influence of electrostatic interactions is negligible, and consequently, the antioxidant or retarding effect cannot be correlated with electrostatic adsorption onto the membrane surface, leading to a local concentration increase as was documented for dopamine and other catecholamines.^60,61^ Instead, the ability to penetrate the lipid bilayer and its orientation within the membrane might be essential.

In terms of protection against oxidative stress, SMe1EC2 has an advantage over its precursor, stobadine, due to its improved bioavailability and lower basicity, which enhances its ability to reduce ROS levels.^34^ Molecular descriptors (see Table S2) showed that, in their neutral form, all three ibogalogs have lower dipole moments (2.4–2.9 D) compared to SMe1EC2 (6.48 D), but similar lipophilicity (log *P* ≈2.6). Even if their tertiary amine becomes protonated at physiological pH, forming an ammonium salt, as suggested by the calculated p*K*_a_ of their conjugate acids (∼10, Table S2), the indole part of the molecule remains lipophilic. This allows the compounds to localize at the lipid/water interface, where they can interact with alkylperoxyl radicals, both those entering from the aqueous phase and those formed within the lipid bilayer during the propagation phase. Other parameters in Table S2 also support a favorable lipophilic/hydrophilic balance, enabling the ibogalogues to function effectively in biological environments and potentially increasing their likelihood of reacting with free radicals in tissues.

Given their structural features and lipophilicity, ibogalogs likely integrate into neuronal synaptic membranes, where they may reduce ROS directly or modulate lipid packing, thereby diminishing the susceptibility of polyunsaturated fatty acid chains to peroxidative attack. Additionally, their ability to limit lipid peroxidation may involve indirect mechanisms, such as the stabilization of membrane-bound antioxidant enzymes or modulation of intracellular redox signaling pathways. As in the case of iboga alkaloids,^59,62^ ibogalogs modulate neurotransmitter activity, especially serotonin,^9^ which can be sensitive to oxidative imbalance. It is therefore plausible that the protective effect toward synaptosomal membranes observed in our study may also involve the restoration of calcium signaling, synaptic function, and overall neurochemical balance, common downstream consequences of oxidative stress in neurons. This multifaceted activity would enhance the modulatory and protective relevance of ibogalogs, particularly in the context of neurodegenerative diseases, including Alzheimer’s and Parkinson’s disease, motor neuron disease, and epilepsy, where oxidative stress and neurochemical dysregulation converge.^63^ Further insights are needed to explain the observed effects and to explore the possible mechanisms of radical trapping by these nonphenolic antioxidants.

## 3 Materials and Methods

The leukocyte-platelet-enriched fraction of blood from mixed donors was obtained from the Regional Center for Blood Donation and Transfusion (Lodz, Poland). Blood donor recruitment was conducted at the Center, according to national legal procedures and European Union regulations (including the regulation (EU) 2016/679 of the European Parliament and of the Council of April 27, 2016 on the protection of natural persons regarding the processing of personal data and on the free movement of such data). As the material was fully anonymized and derived from postdonation medical waste, no additional approval from Regional Ethical Committees was required. 2,2′-Azobis­(2-amidinepropane) dihydrochloride (AAPH) was purchased from Sigma-Aldrich (Darmstadt, Germany). EDTA, EGTA, and phenylmethylsulfonyl fluoride (PMSF) were purchased from Sigma-Aldrich (St. Louis, MO, USA). The BCA protein assay kit was obtained from Thermo Fisher Scientific (Pierce BCA Protein Assay Kits, Waltham, MA, USA). TBG fumarate, IBG hydrochloride, and DM506 hydrochloride (Figure 1) were synthesized by Ambeed, Inc. (Arlington Hts, IL, USA) according to previous protocols.^10,64^ Salts and solvents were purchased from commercial suppliers and used as received.

### 3.1 Hemolysis Measurements

The leukocyte-platelet-enriched fraction of human blood was centrifuged at 2,500*g* for 5 min at 4 °C to remove the plasma and leukocyte layers. The hematocrit of the erythrocytes was determined using the microcapillary method. Cells were diluted in suspension buffer (140 mM NaCl, 10 mM sodium phosphate, pH = 7.4) to a hematocrit of 2%. To determine the % hemolysis, erythrocytes were incubated (37 °C) for 1 and 2 h with 50 mM AAPH, in the absence and presence of TBG, IBG, or DM506 (0.01–10 μM), and subjected to processing according to a previous method.^65^ We used this concentration range based on previous results, indicating that ibogalogs activate the 5-HT_2A_ receptor at nanomolar concentrations and inhibit the α7 nicotinic acetylcholine receptor (nAChR) at micromolar concentrations.^6,7,10,64,66^

### 3.2 Measurement of Potassium Leakage

Following drug treatment, erythrocyte suspensions were centrifuged at 825*g* for 10 min, and the resulting supernatant was analyzed for K^+^ content using flame atomic emission spectrometry,^23^ utilizing a SpectrAA-300 instrument (Varian, Australia). Results were expressed in parts per million (ppm).

### 3.3 Preparation of Erythrocyte Ghosts

Erythrocyte ghosts were prepared according to the method of Dodge et al.^67^ Briefly, erythrocytes were hemolyzed in 20 mM phosphate buffer (pH 7.4), containing 1 mM EDTA, 1 mM EGTA, and 0.5 mM PMSF (serine protease inhibitor). The resulting ghosts were then washed sequentially with 20, 10, and 5 mM ice-cold phosphate buffer (pH 7.4) until the ghosts were free of residual hemoglobin. The protein content in the membrane preparations was determined by using the BCA protein assay.

### 3.4 Determination of Thiobarbituric Acid-Reactive Substances in Erythrocyte Ghosts Membranes and Synaptosomal Fractions

The extent of lipid peroxidation in erythrocyte ghost membranes and synaptosomal fractions was assessed by measuring the levels of TBARS, following the same protocol for both sample types. After incubation with AAPH in the presence or absence of the ibogalog tested, each sample was treated with 1% (w/v) phosphoric acid and 0.6% (w/v) thiobarbituric acid solutions, both prepared in 0.1 M HCl. The suspensions were then heated in a water bath for 45 min and subsequently cooled on ice. TBARS were extracted by adding *n*-butanol to each sample, followed by thorough mixing. The samples were allowed to separate into aqueous and organic phases for 90 min. The absorbance of the *n*-butanol layer was measured at 532 nm. The obtained absorbance values were used as an index of lipid peroxidation and expressed as absorbance units per milligram of protein (TBARS/mg of protein).

### 3.5 Extraction of Synaptosomes from the Hippocampus and Frontal Cortex of a Rat Brain

All experimental procedures were approved by the Regional Ethics Committee for Animal Experimentation (permission number: 1/LB122/2019), carried out in accordance with the European Communities Council Directive (86/609/EEC and 2010/63/EU). The experimental design and procedures followed the ARRIVE 2.0 guidelines. Four male Wistar rats (3 months, 250–300 g) were obtained from Charles River Laboratories (Gottingen, Germany). Synaptosomal fractions isolated from the same four rats were equally divided and subjected to all experimental conditions (within-subject design). Data are presented as the mean of n = 4 biological replicates *per* condition. Animals were housed in standard cages in groups with ad libitum access to standard laboratory chow and tap water. They were maintained under controlled environmental conditions: temperature of 22 ± 1 °C and a 12 h light/dark cycle (lights on from 8:00 a.m. to 8:00 p.m.). Efforts were made to minimize animal suffering and reduce the number of animals used. Animals were anesthetized with isoflurane (Aerrane, Baxter, UK) and euthanized by decapitation. Brains were rapidly removed, and hippocampal formation (HPC) along with frontal cortex (FC) was dissected bilaterally on ice. Tissues were immediately processed using the Minute Synaptosome Isolation Kit (Invent Biotechnologies, Plymouth, MN, USA) for synaptosome fraction isolation.

### 3.6 Western Blotting

To verify the successful isolation of synaptosomes, synaptophysin levels were analyzed using Western blotting. A total of 5 μg of protein from synaptosomal samples of both HPC and FC of rat brains was loaded onto polyacrylamide gels, and SDS-PAGE was performed. PageRuler Plus Prestained Protein Ladder (Thermo Fisher, Waltham, Massachusetts, USA) was loaded alongside the samples to verify the molecular weight (∼38 kDa) of the target protein. Following electrophoresis, a wet transfer to a poly­(vinylidene fluoride) (PVDF) membrane was carried out. The membrane was then washed three times with TBST buffer (10 mM Tris, pH 8.0, 150 mM NaCl, and 0.5% Tween 20) for 5 min at room temperature (*RT*) with agitation. Blocking was performed by incubating the membrane in 5% nonfat milk dissolved in TBST for 1 h at RT with agitation. Subsequently, the membrane was washed three times with TBST and incubated with the primary antibody: anti-Synaptophysin (dilution 1:1000, #4329, Cell Signaling, Danvers, MA, USA), prepared according to the manufacturer’s instructions. Incubation was carried out overnight at 4 °C with agitation. After three washes with TBST, the membrane was incubated with the secondary antibody: HRP-linked antirabbit IgG (dilution 1:1000, #7074, Cell Signaling, Danvers, MA, USA) for 1 h at RT with agitation. The chemiluminescent signal was visualized using an Azure 300 Chemiluminescence Imager (Azure Biosystems, Sierra Trinity, USA) with the SuperSignal West Pico PLUS substrate (Thermo Fisher, Waltham, Massachusetts, USA).

### 3.7 Statistical Analysis

Prism software (GraphPad 5.0, Software Inc., La Jolla, CA, USA) was used for the statistical analysis of the results and for the concentration–response curves. Test results were analyzed using one-way ANOVA and Tukey’s multiple comparison posthoc test. Values of *p* < 0.05 are considered statistically significant. Cohen’s *d* was used as a standardized measure of effect size, with values of 0.2, 0.5, and ≥0.8 indicating small, medium, and large effects, respectively. For all comparisons, 95% confidence intervals (CIs) were calculated and used to support the interpretation of the differences between groups.

### 3.8 Preparation of Liposomes

Large unilamellar vesicles (LUVs) were prepared from multilamellar vesicles (MLVs) using the extrusion procedure described previously.^68,69^ The preparation began by dissolving 65.3 mg of dimyristoylphosphatidylcholine (DMPC) in a small amount of chloroform (∼1.5 mL). After the solution was mixed for 1 min in a vortex, 4 μL of methyl linoleate was added, and the mixture was vortexed for another minute. The chloroform was then slowly evaporated by using a rotary evaporator, forming a thin lipid layer on the inner walls of the flask. The flasks containing the lipid layers were left to dry overnight at RT under vacuum. The dried liposomes were suspended in 4.4 mL of PBS buffer. The final lipid concentrations were 2.74 mM methyl linoleate and 20.2 mM DMPC. The liposome suspension was extruded 21 times through a 0.1 μm pore membrane using an Avanti mini-extruder (Avanti Polar Lipids Inc.). The size distribution of the LUVs was determined by DLS (Dynamic Light Scattering), with an average size of 170 ± 45 nm (consistent with previously prepared LUVs).^68^

### 3.9 Antioxidant Activity of Ibogalogs in Model Lipid Systems

Oxygen consumption during lipid peroxidation was measured at 37 °C by using a Yellow Spring Instrument 5300A biological oxygen monitor (YSI, Yellow Springs, OH, USA) connected to two Clark-type oxygen electrodes in a temperature-controlled water bath. A 2 mL sample of the liposome system was placed in a small chamber equipped with a magnetic stirrer, and the electrode was inserted into the chamber. Peroxidation was initiated by injecting a 0.2 M aqueous solution of AAPH. When the oxygen content dropped to 90–80% of the initial value, 10 μL of antioxidant solution (0.2 mM PMHC in ethanol or 2 mM ibogalog in water) was added using a glass microsyringe. The final concentrations of the components in the samples were as follows: 2.74 mM methyl linoleate, 10 mM AAPH, 10 μM of each ibogalog, and 1 μM PMHC (2,2,5,7,8-pentamethylchroman-6-ol, reference antioxidant).

### 3.10 Molecular Descriptors for Ibogalogs and Known Antioxidants

Different molecular descriptors were calculated for ibogalogs and known antioxidants such as PMHC, stobadine, and its derivative SMe1EC2 ^34–37^ (see molecular structures in Figure 1), using QikProp software (Schrödinger Suite 2020-3). Molecular descriptors included the partition coefficient between water and octanol (LogP), brain/blood partition coefficient (LogBB), volume (Vol), polar surface area, solvent-accessible surface area (SASA), hydrophobic [i.e., saturated carbon (C) and attached hydrogen (H)] (FOSA), hydrophilic (i.e., N, O, and H on heteroatoms) (FISA), and π (C and attached H) (PISA) components of SASA, molecular polarizability, rotatable bonds (number of nontrivial, not hindered—not alkene, amide, small ring—rotatable bonds), dipole moment, dipole^2^/Vol, ionization potential (negative of HOMO energy), electron affinity (negative of LUMO energy), globularity (sphere globularity = 1), and H-bond donors and acceptors.

### 3.11 Computational Details

Weak X–H (*X* = N,C) bond dissociation enthalpies were calculated using the ACP approach described in ref 70. Briefly, the approach uses the ωB97XD density functional^71^ with double-ζ pcseg-1 basis sets^72^ augmented with ACPs that elevate the performance of the method to within a mean absolute error of 0.7 kcal/mol of those obtained with (RO)­CBS-QB3 ^73^ but with no increase in computational time over uncorrected ωB97XD.

Each parent molecule and the corresponding radical structure were subjected to conformational searches using the GFNFF force field via the CREST program.^74^ The number of structures generated for each species ranged from 4 (stobadine) to 265 (SMe1EC2). All generated structures were subjected to full geometry optimizations using ωB97XD/pcseg-1-ACP in implicit solvents (hexene—to simulate a bilayer environment—and water) using the SMD solvent model^75^ and without an implicit solvent (i.e., gas phase). The lowest energy structures obtained were subjected to frequency calculations. All calculated frequencies were verified as being positive and were used unscaled to obtain the thermal corrections to molecular and radical enthalpies to assess bond dissociation enthalpies (BDEs).

Vertical ionization potentials (IPs) were computed for the minimum energy conformer for each of the parent structures using the M06-2*X* functional^76^ with 6–311 + G­(d,p) basis sets. Electronic energies were used to obtain only the IPs. All quantum chemical calculations were performed using the Gaussian-16 package.^77^

## 4 Conclusions

This study provides novel insight into the redox-modulatory properties of ibogalogs, linking their known pharmacological effects with membrane-protective antioxidant capacities. All three ibogalogs protected human erythrocytes from oxidative damage by preventing hemolysis, potassium leakage, and reducing MDA levels, a marker of lipid peroxidation. These effects were particularly pronounced for TBG. Importantly, none of the tested compounds induced hemolysis at concentrations ranging from 0.01 to 10 μM, indicating a membrane safety profile. In rat hippocampal and cortical synaptosomal fractions, ibogalogs exerted protective effects under oxidative stress, suggesting that their membrane-stabilizing and antioxidant properties may extend to neural components. Among the tested compounds, TBG showed the strongest antioxidant performance in liposomal systems, particularly under lipid peroxyl radical-mediated peroxidation. In contrast, DM506 and IBG were less effective, primarily retarding autoxidation without a clear induction period. Quantum chemical calculations support the experimental observation that the position and nature of indole substituents, especially the methoxy group, critically influence bond dissociation enthalpies, radical delocalization, and antioxidant efficacy. This supports a dual mechanism of action for ibogalogs: in addition to modulating 5-HT transmission,^6–10^ they may exert antioxidant activity through direct lipid interaction and radical scavenging. Their lipophilic character likely facilitates integration into biological membranes, enabling them to reduce oxidative damage directly or alter lipid packing or order, as observed with phytosterols and polyphenols,^78,79^ to decrease vulnerability to peroxidative damage. This suggests neuromodulatory and neuroprotective potential of ibogalogs, meriting further studies to elucidate their mechanisms of action to establish whether these effects are translatable in vivo.

## 5 Limitations of the Study

This study is limited to in vitro and ex vivo models, which provide mechanistic insight but do not reflect the full complexity of in vivo systems. Although the translational relevance of tested ibogalogs remains speculative, behavioral results in mice showed that ibogalogs decrease pain and inflammation associated with potassium superoxide, a radical that induces oxidative stress (manuscript in preparation). While quantum chemical modeling supports our interpretation of antioxidant efficiency, these predictions require further experimental validation. Future studies should include animal models to evaluate the neuroprotective efficacy and pharmacodynamic properties. In addition, mechanistic investigations, including selective structural modifications, such as replacing the indole N–H group with N–D or N–CH_3_, may help clarify hydrogen donation mechanisms and radical stabilization pathways.

## Supplementary Material



## Notes

Mulliken spin densities at various atomic centers in the DM506, TBG, and IBG radicals (Table S1), plot of selected Mulliken spin densities for the radicals of DM506, TBG, and IBG (Figure S1), and molecular descriptors for ibogalogs and known antioxidants (Table S2) (PDF)

## Abbreviations

5-HT: 5-hydroxytryptamine (serotonin)
5-HT_2A_: serotonin receptor subtype 2A
DM506 (ibogaminalog): 3-methyl-1,2,3,4,5,6-hexahydroazepino­[4,5-*b*]­indole
TBG: (tabernanthalog), 8-methoxy-3-methyl-1,2,3,4,5,6-hexahydroazepino­[4,5-*b*]­indole fumarate
IBG: (ibogainalog), 9-methoxy-3-methyl-1,2,3,4,5,6-hexahydroazepino­[4,5-*b*]­indole hydrochloride
AAPH: 2,2′-azobis (2-amidinopropane) dihydrochloride
TBARS: thiobarbituric acid-reactive substances
ROS: reactive oxygen species; stobadine, (−)-cis-2,8-dimethyl-2,3,4,4a,5,9b-hexahydro-1H-pyrido­[4,3*b*]­indole
SMe1EC2: (±)-cis-8-methoxy-2,3,4,4a,5,9b-hexahydro-1H-pyrido­[4,3-*b*]­indole-2-carboxylic acid ethyl ester
*RT*: room temperature
Log *P*: lipid solubility
LogBB: brain/blood partition coefficient
Vol: molecular volume
PSA: polar surface area
SASA: solvent-accessible surface area
FOSA: hydrophobic component of SASA
FISA: hydrophilic component of SASA
PISA: π component of SASA
H-bond: hydrogen bond

## References

[1] Sayre L. M., Perry G., Smith M. A. (2008). Oxidative Stress and Neurotoxicity. Chem. Res. Toxicol..

[2] Jodko-Piórecka K., Litwinienko G. (2015). Antioxidant
Activity of Dopamine and L-DOPA in Lipid
Micelles and Their Cooperation with an Analogue of α-Tocopherol. Free Radical Biol. Med..

[3] Peña-Bautista C., Vento M., Baquero M., Cháfer-Pericás C. (2019). Lipid Peroxidation
in Neurodegeneration. Clin. Chim. Acta.

[4] Lleó A., Greenberg S. M., Growdon J. H. (2006). Current Pharmacotherapy for Alzheimer’s
Disease. Annu. Rev. Med..

[5] Kozlov A. V., Javadov S., Sommer N. (2024). Cellular ROS
and Antioxidants: Physiological
and Pathological Role. Antioxidants.

[6] Arias H. R., Micheli L., Rudin D., Bento O., Borsdorf S., Ciampi C., Marin P., Ponimaskin E., Manetti D., Romanelli M. N., Ghelardini C., Liechti M. E., Di Cesare Mannelli L. (2024). Non-Hallucinogenic Compounds Derived
from Iboga Alkaloids Alleviate Neuropathic and Visceral Pain in Mice
through a Mechanism Involving 5-HT_2A_ Receptor Activation. Biomed. Pharmacother..

[7] Arias H. R., Rudin D., Hines D. J., Contreras A., Gulsevin A., Manetti D., Anouar Y., De Deurwaerdere P., Meiler J., Romanelli M. N., Liechti M. E., Chagraoui A. (2024). The Novel
Non-Hallucinogenic Compound DM506 (3-Methyl-1,2,3,4,5,6-Hexahydroazepino­[4,5-*b*]­Indole) Induces Sedative- and Anxiolytic-like Activity
in Mice by a Mechanism Involving 5-HT_2A_ Receptor Activation. Eur. J. Pharmacol..

[8] Arias H. R., Rudin D., Luethi D., Valenta J., Leśniak A., Czartoryska Z., Olejarz-Maciej A., Doroz-Płonka A., Manetti D., De Deurwaerdère P., Romanelli M. N., Handzlik J., Liechti M. E., Chagraoui A. (2025). The Psychoplastogens
Ibogaminalog and Ibogainalog Induce Antidepressant-like Activity in
Naïve and Depressed Mice by Mechanisms Involving 5-HT_2A_ Receptor Activation and Serotonergic Transmission. Prog. Neuropsychopharmacol Biol. Psychiatry.

[9] Arias H. R., Micheli L., Jensen A. A., Galant S., Vandermoere F., Venturi D., Manetti D., Romanelli M. N., Ghelardini C., Marin P., Di Cesare Mannelli L. (2025). Ibogalogs
Decrease Neuropathic Pain in Mice through a Mechanism Involving Crosstalk
between 5-HT_2A_ and mGlu_2_ Receptors. Biomed. Pharmacother..

[10] Cameron L. P., Tombari R. J., Lu J., Pell A. J., Hurley Z. Q., Ehinger Y., Vargas M. V., McCarroll M. N., Taylor J. C., Myers-Turnbull D., Liu T., Yaghoobi B., Laskowski L. J., Anderson E. I., Zhang G., Viswanathan J., Brown B. M., Tjia M., Dunlap L. E., Rabow Z. T., Fiehn O., Wulff H., McCorvy J. D., Lein P. J., Kokel D., Ron D., Peters J., Zuo Y., Olson D. E. (2021). A Non-Hallucinogenic Psychedelic Analogue with Therapeutic
Potential. Nature.

[11] Looschen K., Khatri S. N., Maulik M., Salisbury C., Carman A. F., Corriveau K., Smith C., Manetti D., Romanelli M. N., Arias H. R., Gipson C. D., Mitra S. (2024). Novel Psychoplastogen
DM506 Reduces Cue-Induced Heroin-Seeking and Inhibits Tonic GABA Currents
in the Prelimbic Cortex. Neurochem. Int..

[12] Lu J., Tjia M., Mullen B., Cao B., Lukasiewicz K., Shah-Morales S., Weiser S., Cameron L. P., Olson D. E., Chen L., Zuo Y. (2021). An Analog of Psychedelics Restores
Functional Neural Circuits Disrupted by Unpredictable Stress. Mol. Psychiatry.

[13] Heinsbroek J. A., Giannotti G., Bonilla J., Olson D. E., Peters J. (2023). Tabernanthalog
Reduces Motivation for Heroin and Alcohol in a Polydrug Use Model. Psychedelic Med..

[14] Paškulin R., Jamnik P., Danevčič T., Koželj G., Krašovec R., Krstić-Milošević D., Blagojević D., Strukelj B. (2012). Metabolic Plasticity and the Energy
Economizing Effect of Ibogaine, the Principal Alkaloid of Tabernanthe
Iboga. J. Ethnopharmacol..

[15] Tatalović N., Vidonja Uzelac T., Oreščanin
Dušić Z., Nikolić-Kokić A., Bresjanac M., Blagojević D. (2021). Ibogaine-Mediated ROS/Antioxidant
Elevation in Isolated
Rat Uterus Is ?-Adrenergic Receptors and K_ATP_ Channels
Mediated. Antioxidants.

[16] Chagraoui A., Haro Santillan L. A., Bocian R., Cohen S. J., Sarmiento
Ruíz Y. E., Knox M., Kazmierska-Grebowska P., Zimoń W., Stackman R. W., Flores-Hernandez J., Arias H. R. (2025). Ibogalogs Improve Spatial and Recognition Memory in
Rodents through a Mechanism Involving 5-HT_2A_ Receptor Activation-Enhanced
NMDA Receptor Activity in Hippocampal Pyramidal CA1 Neurons. Biomed. Pharmacother..

[17] Ali S., Tian X., Cunningham K. A., Zhou J. (2025). Old Dog, New Tricks:
Ibogaine and Its Analogs as Potential Neurotherapeutics. J. Med. Chem..

[18] Nikolić-Kokić A., Oreščanin-Dušić Z., Spasojević I., Slavić M., Mijušković A., Paškulin R., Miljević C. ˇ., Spasić M. B., Blagojević D. P. (2015). Ex Vivo Effects of Ibogaine on the Activity of Antioxidative
Enzymes in Human Erythrocytes. J. Ethnopharmacol..

[19] Yin H., Xu L., Porter N. A. (2011). Free Radical
Lipid Peroxidation: Mechanisms and Analysis. Chem. Rev..

[20] Barnham K. J., Masters C. L., Bush A. I. (2004). Neurodegenerative
Diseases and Oxidative
Stress. Nat. Rev. Drug Discovery.

[21] Castellanos D. B., Martín-Jiménez C. A., Rojas-Rodríguez F., Barreto G. E., González J. (2021). Brain Lipidomics as a Rising Field
in Neurodegenerative Contexts: Perspectives with Machine Learning
Approaches. Front Neuroendocrinol.

[22] Wong M. W., Braidy N., Poljak A., Pickford R., Thambisetty M., Sachdev P. S. (2017). Dysregulation of Lipids in Alzheimer’s
Disease
and Their Role as Potential Biomarkers. Alzheimers
Dement.

[23] Remigante A., Studzian M., Spinelli S., Piotrowski P., Litwinienko G., Gorny K., Raczynski P., Marino A., Morabito R., Grebowski J. (2025). Metallofullerenol
Gd@C_82_(OH)_22_ Preserves Human Erythrocyte Plasma
Membrane Integrity from AAPH-Induced Oxidative Stress: Molecular Mechanisms
and Antioxidant Activity. Free Radical Biol.
Med..

[24] Grebowski J., Studzian M., Lekki-Porebski S., Konarska A., Wolszczak M., Litwinienko G., Pulaski L. (2026). Metallofullerenol Sc_3_N@C_80_(OH)_18_: A New Generation Radioprotector Protecting
Human Erythrocytes Against Multiple Biochemical Damage Modes Upon
Gamma Irradiation, Identifying It as a Scavenger of Short- and Long-Lived
Radicals. Adv. Healthcare Mater..

[25] Grebowski J., Konopko A., Krokosz A., DiLabio G. A., Litwinienko G. (2020). Antioxidant
Activity of Highly Hydroxylated Fullerene C_60_and Its Interactions
with the Analogue of α-Tocopherol. Free
Radical Biol. Med..

[26] Stanzani A., Sansone A., Brenna C., Baldassarro V. A., Alastra G., Lorenzini L., Chatgilialoglu C., Laface I., Ferreri C., Neri L. M., Calzà L. (2023). Erythrocyte
Plasma Membrane Lipid Composition Mirrors That of Neurons and Glial
Cells in Murine Experimental In Vitro and In Vivo Inflammation. Cells.

[27] Berlin E., Lork A. A., Ernst C., Fletcher J. S., Phan N. T. N. (2025). Plasma
Membrane Lipid Composition and Turnover in Human Midbrain Neurons
Investigated by Time-of-Flight Mass Spectrometry. Biomolecules.

[28] Ali O., Szabó A. (2023). Review of Eukaryote Cellular Membrane Lipid Composition,
with Special Attention to the Fatty Acids. Int.
J. Mol. Sci..

[29] Grebowski J., Konarska A., Piotrowski P., Wolszczak M., Litwinienko G. (2024). Kinetics of Metallofullerenol Reactions
with the Products
of Water Radiolysis: Implications for Radiotherapeutics. ACS Appl. Nano Mater..

[30] Grebowski J., Kazmierska-Grebowska P., Cichon N., Konarska A., Wolszczak M., Litwinienko G. (2022). Fullerenol C_60_(OH)_36_ Protects
the Antioxidant Enzymes in Human Erythrocytes against Oxidative Damage
Induced by High-Energy Electrons. Int. J. Mol.
Sci..

[31] Chen X., Guo C., Kong J. (2012). Oxidative Stress in
Neurodegenerative Diseases. Neural Regener.
Res..

[32] Viola T. W., Orso R., Florian L. F., Garcia M. G., Gomes M. G. S., Mardini E. M., Niederauer J. P. O., Zaparte A., Grassi-Oliveira R. (2023). Effects of
Substance Use Disorder on Oxidative and Antioxidative Stress Markers:
A Systematic Review and Meta-Analysis. Addict.
Biol..

[33] Aguiar C. C. T., Almeida A. B., Araújo P. V. P., Abreu R. N. D. C. d., Chaves E. M. C., Vale O. C. d., Macêdo D. S., Woods D. J., Fonteles M. M. d. F., Vasconcelos S. M. M. (2012). Oxidative
Stress and Epilepsy: Literature Review. Oxid.
Med. Cell. Longevity.

[34] Balcerczyk A., Bartosz G., Drzewinska J., Piotrowski Ł., Pulaski Ł., Stefek M. (2014). Antioxidant Action
of SMe1EC2, the Low-Basicity Derivative of the Pyridoindole Stobadine,
in Cell Free Chemical Models and at Cellular Level. Interdiscip. Toxicol..

[35] Stolc S., Snirc V., Májeková M., Gáspárová Z., Gajdosíková A., Stvrtina S. (2006). Development of the
New Group of Indole-Derived Neuroprotective Drugs Affecting Oxidative
Stress. Cell. Mol. Neurobiol..

[36] Štolc S., Vlkolinský R., Pavlásek J. (1997). Neuroprotection by the Pyridoindole
Stobadine: A Minireview. Brain Res. Bull..

[37] Vincenzi F. F., Hinds T. R. (1999). Stobadine: Bellwether
of a Broader View of Drug Actions. Life Sci..

[38] Grebowski J., Krokosz A. (2015). The Effect of Highly
Hydroxylated Fullerenol C_60_(OH)_36_ on Human Erythrocyte
Membrane Organization. J. Spectrosc..

[39] Remigante A., Spinelli S., Gambardella L., Bozzuto G., Vona R., Caruso D., Villari V., Cappello T., Maisano M., Dossena S., Marino A., Morabito R., Straface E. (2024). Internalization
of Nano- and Micro-Plastics in Human Erythrocytes Leads to Oxidative
Stress and Estrogen Receptor-Mediated Cellular Responses. Free Radical Biol. Med..

[40] Delorenzi J. C., Freire-de-Lima L., Gattass C. R., de Andrade Costa D., He L., Kuehne M. E., Saraiva E. M. B. (2002). In Vitro Activities of Iboga Alkaloid
Congeners Coronaridine and 18-Methoxycoronaridine against Leishmania
Amazonensis. Antimicrob. Agents Chemother..

[41] Havel V., Kruegel A. C., Bechand B., McIntosh S., Stallings L., Hodges A., Wulf M. G., Nelson M., Hunkele A., Ansonoff M., Pintar J. E., Hwu C., Ople R. S., Abi-Gerges N., Zaidi S. A., Katritch V., Yang M., Javitch J. A., Majumdar S., Hemby S. E., Sames D. (2024). Oxa-Iboga
Alkaloids Lack Cardiac Risk and Disrupt Opioid Use in Animal Models. Nat. Commun..

[42] Tatalović N., Vidonja Uzelac T., Mijović M., Koželj G., Nikolić-Kokić A., Oreščanin
Dušić Z., Bresjanac M., Blagojević D. (2022). Ibogaine Has
Sex-Specific Plasma Bioavailability, Histopathological and Redox/Antioxidant
Effects in Rat Liver and Kidneys: A Study on Females. Life.

[43] Radosinska J., Vrbjar N. (2021). Erythrocyte Deformability and Na,
K-ATPase Activity
in Various Pathophysiological Situations and Their Protection by Selected
Nutritional Antioxidants in Humans. Int. J.
Mol. Sci..

[44] Boligon, A. A. ; Piana, M. ; Schawnz, T. G. ; Pereira, R. P. ; Rocha, J. B. T. ; Athayde, M. L. Chromatographic Analysis and Antioxidant Capacity of *Tabernaemontana Catharinensis* . Nat. Prod. Commun. 2014, 9 1934578X1400900119 10.1177/1934578X1400900119. 24660464

[45] Vidonja
Uzelac T., Tatalović N., Mijović M., Miler M., Grahovac T., Oreščanin
Dušić Z., Nikolić-Kokić A., Blagojević D. (2024). Ibogaine Induces Cardiotoxic Necrosis in Rats The Role
of Redox Processes. Int. J. Mol. Sci..

[46] Venkateshappa C., Harish G., Mahadevan A., Srinivas Bharath M. M., Shankar S. K. (2012). Elevated Oxidative Stress and Decreased Antioxidant
Function in the Human Hippocampus and Frontal Cortex with Increasing
Age: Implications for Neurodegeneration in Alzheimer’s Disease. Neurochem. Res..

[47] Zlatković J., Todorović N., Bošković M., Pajović S. B., Demajo M., Filipović D. (2014). Different Susceptibility of Prefrontal
Cortex and Hippocampus to Oxidative Stress Following Chronic Social
Isolation Stress. Mol. Cell. Biochem..

[48] Preston A. R., Eichenbaum H. (2013). Interplay
of Hippocampus and Prefrontal Cortex in Memory. Curr. Biol..

[49] Kazmierska-Grebowska P., Jankowski M. M., MacIver M. B. (2024). Missing Puzzle Pieces in Dementia
Research: HCN Channels and Theta Oscillations. Aging Dis.

[50] Pardillo-Díaz R., Pérez-García P., Castro C., Nunez-Abades P., Carrascal L. (2022). Oxidative
Stress as a Potential Mechanism Underlying
Membrane Hyperexcitability in Neurodegenerative Diseases. Antioxidants.

[51] Ebding J., Mazzone F., Kins S., Pielage J., Maritzen T. (2025). How Neurons
Cope with Oxidative Stress. Biol. Chem..

[52] He J., Chen Y., Dai S., Chen F., Wang Y., Shi T., Chen L., Liu Y., Chen J., Xie P. (2023). First Insights
into Region-Specific Lipidome Alterations of Prefrontal Cortex and
Hippocampus of Mice Exposed Chronically to Microcystins. Environ. Int..

[53] Jodko-Piórecka, K. ; Cedrowski, J. ; Litwinienko, G. Physico-Chemical Principles of Antioxidant Action, Including Solvent and Matrix Dependence and Interfacial Phenomena. In Measurement of Antioxidant Activity & Capacity; John Wiley & Sons, Ltd, 2018; pp 225–272.

[54] Mrvová N., Škandík M., Bezek Š., Sedláčková N., Mach M., Gaspárová Z., Luptáková D., Padej I., Račková L. (2017). Pyridoindole SMe1EC2
as Cognition Enhancer in Ageing-Related Cognitive Decline. Interdiscip. Toxicol..

[55] Bezek Š., Brnoliaková Z., Sotníková R., Knezl V., Paulovičová E., Navarová J., Bauer V. (2017). Monotherapy of Experimental Metabolic
Syndrome: I. Efficacy and Safety. Interdiscip.
Toxicol..

[56] Mulder P., Litwinienko G., Lin S., MacLean P. D., Barclay L. R. C., Ingold K. U. (2006). The L-Type Calcium
Channel Blockers, Hantzsch 1,4-Dihydropyridines,
Are Not Peroxyl Radical-Trapping, Chain-Breaking Antioxidants. Chem. Res. Toxicol..

[57] Chepelev L. L., Beshara C. S., MacLean P. D., Hatfield G. L., Rand A. A., Thompson A., Wright J. S., Barclay L. R. C. (2006). Polypyrroles
as Antioxidants: Kinetic Studies on Reactions of Bilirubin and Biliverdin
Dimethyl Esters and Synthetic Model Compounds with Peroxyl Radicals
in Solution. Chemical Calculations on Selected Typical Structures. J. Org. Chem..

[58] Zielinski Z. A. M., Pratt D. A. (2017). Lipid Peroxidation:
Kinetics, Mechanisms, and Products. J. Org.
Chem..

[59] Galano A., Reiter R. J. (2018). Melatonin and Its
Metabolites vs Oxidative Stress:
From Individual Actions to Collective Protection. J. Pineal Res..

[60] Jodko-Piorecka K., Litwinienko G. (2013). First Experimental
Evidence of Dopamine Interactions
with Negatively Charged Model Biomembranes. ACS Chem. Neurosci..

[61] Jodko-Piórecka K., Sikora B., Kluzek M., Przybylski P., Litwinienko G. (2022). Antiradical
Activity of Dopamine, L-DOPA, Adrenaline,
and Noradrenaline in Water/Methanol and in Liposomal Systems. J. Org. Chem..

[62] Glick S. D., Kuehne M. E., Raucci J., Wilson T. E., Larson D., Keller R. W., Carlson J. N. (1994). Effects of *Iboga* Alkaloids on Morphine and Cocaine Self-Administration in Rats: Relationship
to Tremorigenic Effects and to Effects on Dopamine Release in Nucleus
Accumbens and Striatum. Brain Res..

[63] Houldsworth A. (2023). Role of Oxidative
Stress in Neurodegenerative Disorders: A Review of Reactive Oxygen
Species and Prevention by Antioxidants. Brain
Commun..

[64] Tae H.-S., Ortells M. O., Tekarli B. J., Manetti D., Romanelli M. N., McIntosh J. M., Adams D. J., Arias H. R. (2023). DM506 (3-Methyl-1,2,3,4,5,6-Hexahydroazepino­[4,5-*b*]­Indole Fumarate), a Novel Derivative of Ibogamine, Inhibits
α7 and α9α10 Nicotinic Acetylcholine Receptors by
Different Allosteric Mechanisms. ACS Chem. Neurosci..

[65] Grebowski J., Kazmierska P., Litwinienko G., Lankoff A., Wolszczak M., Krokosz A. (2018). Fullerenol C_60_(OH)_36_ Protects
Human Erythrocyte Membrane against High-Energy Electrons. Biochim Biophys Acta Biomembr.

[66] Tae H.-S., Ortells M. O., Yousuf A., Xu S. Q., Akk G., Adams D. J., Arias H. R. (2024). Tabernanthalog and Ibogainalog Inhibit
the α7 and α9α10 Nicotinic Acetylcholine Receptors
via Different Mechanisms and with Higher Potency than the GABA_A_ Receptor and Ca_V_2.2 Channel. Biochem. Pharmacol..

[67] Dodge J. T., Mitchell C., Hanahan D. J. (1963). The Preparation
and Chemical Characteristics
of Hemoglobin-Free Ghosts of Human Erythrocytes. Arch. Biochem. Biophys..

[68] Konopko A., Kusio J., Litwinienko G. (2020). Antioxidant
Activity of Metal Nanoparticles
Coated with Tocopherol-Like Residues The Importance of Studies in
Homo- and Heterogeneous Systems. Antioxidants.

[69] Przybylski P., Żebrowski M., Witkowski W., Cybularczyk-Cecotka M., Litwinienko G. (2024). Antioxidant Activity of Bilirubin in Micellar and Liposomal
Systems Is pH-Dependent. Antioxidants.

[70] Prasad V. K., Otero-de-la-Roza A., DiLabio G. A. (2024). Bridging the Gap between High-Level
Quantum Chemical Methods and Deep Learning Models. Mach. Learn.: Sci. Technol..

[71] Chai J.-D., Head-Gordon M. (2008). Long-Range
Corrected Hybrid Density Functionals with
Damped Atom-Atom Dispersion Corrections. Phys.
Chem. Chem. Phys..

[72] Jensen F. (2014). Unifying General
and Segmented Contracted Basis Sets. Segmented Polarization Consistent
Basis Sets. J. Chem. Theory Comput..

[73] Wood G. P. F., Radom L., Petersson G. A., Barnes E. C., Frisch M. J., Montgomery J. A. (2006). A Restricted-Open-Shell Complete-Basis-Set Model Chemistry. J. Chem. Phys..

[74] Pracht P., Grimme S., Bannwarth C., Bohle F., Ehlert S., Feldmann G., Gorges J., Müller M., Neudecker T., Plett C., Spicher S., Steinbach P., Wesołowski P. A., Zeller F. (2024). CREST-A Program for
the Exploration
of Low-Energy Molecular Chemical Space. J. Chem.
Phys..

[75] Marenich A. V., Cramer C. J., Truhlar D. G. (2009). Universal Solvation Model Based on
Solute Electron Density and on a Continuum Model of the Solvent Defined
by the Bulk Dielectric Constant and Atomic Surface Tensions. J. Phys. Chem. B.

[76] Zhao Y., Truhlar D. G. (2008). The M06 Suite of
Density Functionals for Main Group
Thermochemistry, Thermochemical Kinetics, Noncovalent Interactions,
Excited States, and Transition Elements: Two New Functionals and Systematic
Testing of Four M06-Class Functionals and 12 Other Functionals. Theor. Chem. Acc..

[77] Frisch, M. J. ; Trucks, G. W. ; Schlegel, H. B. ; Scuseria, G. E. ; Robb, M. A. ; Cheeseman, J. R. ; Scalmani, G. ; Barone, V. ; Petersson, G. A. ; Nakatsuji, H. ; Li, X. ; Caricato, M. ; Marenich, A. V. ; Bloino, J. ; Janesko, B. G. ; Gomperts, R. ; Mennucci, B. ; Hratchian, H. P. ; Ortiz, J. V. ; Izmaylov, A. F. ; Sonnenberg, J. L. ; Williams-Young, D. ; Ding, F. ; Lipparini, F. ; Egidi, F. ; Goings, J. ; Peng, B. ; Petrone, A. ; Henderson, T. ; Ranasinghe, D. ; Zakrzewski, V. G. ; Gao, J. ; Rega, N. ; Zheng, G. ; Liang, W. ; Hada, M. ; Ehara, M. ; Toyota, K. ; Fukuda, R. ; Hasegawa, J. ; Ishida, M. ; Nakajima, T. ; Honda, Y. ; Kitao, O. ; Nakai, H. ; Vreven, T. ; Throssell, K. ; Montgomery Jr, J. A. ; Peralta, J. E. ; Ogliaro, F. ; Bearpark, M. J. ; Heyd, J. J. ; Brothers, E. N. ; Kudin, K. N. ; Staroverov, V. N. ; Keith, T. A. ; Kobayashi, R. ; Normand, J. ; Raghavachari, K. ; Rendell, A. P. ; Burant, J. C. ; Iyengar, S. S. ; Tomasi, J. ; Cossi, M. ; Millam, J. M. ; Klene, M. ; Adamo, C. ; Cammi, R. ; Ochterski, J. W. ; Martin, R. L. ; Morokuma, K. ; Farkas, O. ; Foresman, J. B. ; Fox, D. J. Gaussian, Inc. Revision C.01 ; Gaussian, Inc., Wallingford CT, 2016.

[78] Veleva R., Topouzova-Hristova T., Kostadinova A., Benkova D., Trendafilova A., Ivanova V., Moskova-Doumanova V., Mladenova K., Doumanov J., Yordanova V., Staneva G. (2025). Flavonoid Glycosides
and Phenolic Acids from Inula Oculus-Christi Modulate Membrane Organization
and Provide Antioxidant Protection. Molecules.

[79] Wang X., Liu Y., Du F., Zong A., Xu T. (2025). Phytosterol-Mediated
Disturbance of the Lipid Packing Order in the Plasma Membrane Regulates
Inflammatory Response. Food Funct..

