# Supplementary material for: Structure-Dependent Antioxidant Activity of Ibogalogs: Impact of Methoxy Group Position on the Protective Activity in Model and Synaptosomal Lipid Membranes
Source: ACS Chem Neurosci. 2026 Mar 17;17(7):1401–14. doi: 10.1021/acschemneuro.6c00087 (PMC13047532; doi:10.1021/acschemneuro.6c00087)
Supplement: Supplementary file 1 [file cn6c00087_si_001.pdf]

SUPPORTING INFORMATION

Structure-dependent antioxidant activity of ibogalogs: impact of methoxy group position on the protective activity in model and synaptosomal lipid membranes

Paulina Kazmierska-Grebowska,[a*] Jacek Grebowski,[b,c] Michał Żebrowski,[d] Gino A. DiLabio,[e] Oskar Ciesielski,[b] Aneta Balcerczyk,[b] Hugo R. Arias,[f] and Grzegorz Litwinienko[d*]

[a] Department of Neurobiology, Faculty of Biology and Environmental Protection, University of Lodz, Pomorska Str. 141/143, 90-236 Lodz, Poland.
[b] Department of Oncobiology and Epigenetics, Faculty of Biology and Environmental Protection, University of Lodz, Pomorska Str.141/143, 90-236 Lodz, Poland.
[c] Military Institute of Medicine - National Research Institute, Szaserow Str.128, 04-141 Warsaw, Poland.
[d] Faculty of Chemistry, University of Warsaw, Pasteura Str.1, 02-093 Warsaw, Poland.
[e] Department of Chemistry, The University of British Columbia, 3247 University Way, Kelowna, British Columbia V6T 1Z4, Canada.
[f] Department of Pharmacology and Physiology, Oklahoma State University, College of Osteopathic Medicine, Tahlequah, Oklahoma 74464, United States.

*Corresponding authors:
Dr. Paulina Kazmierska-Grebowska, Department of Neurobiology, Faculty of Biology and Environmental Protection, University of Lodz, Pomorska Str. 141/143, 90-236 Lodz, Poland.
E-mail: paulina.kazmierska@biol.uni.lodz.pl.
Prof. Grzegorz Litwinienko, Faculty of Chemistry, University of Warsaw, Pasteura Str. 1, 02-093 Warsaw, Poland.
E-mail: litwin@chem.uw.edu.pl.

Table S1. Mulliken spin densities at various atomic centers in the DM506, TBG, and IBG radicals formed from the scission of the N-H bonds.

Figure S1. Plot of selected Mulliken spin densities for the radicals of DM506, TBG, and IBG formed from the scission of the N-H bond.

Table S2. Molecular descriptors for ibogalogs and known antioxidants.

Table S1. Mulliken spin densities at various atomic centers in the DM506, TBG, and IBG radicals formed from the scission of the N-H bonds.

General structure	Mulliken Spin (e)			
	Center	DM506	TBG	IBG
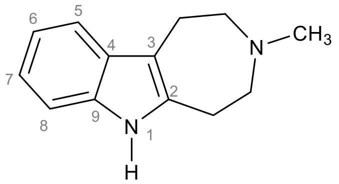	1	0.223	0.193	0.247
	2	-0.048	0.030	-0.115
	3	0.657	0.577	0.679
	4	-0.189	-0.184	-0.169
	5	0.223	0.193	0.247
	6	-0.109	-0.123	-0.059
	7	0.213	0.214	0.127
	8	-0.076	-0.071	-0.029
	9	0.142	0.192	0.065

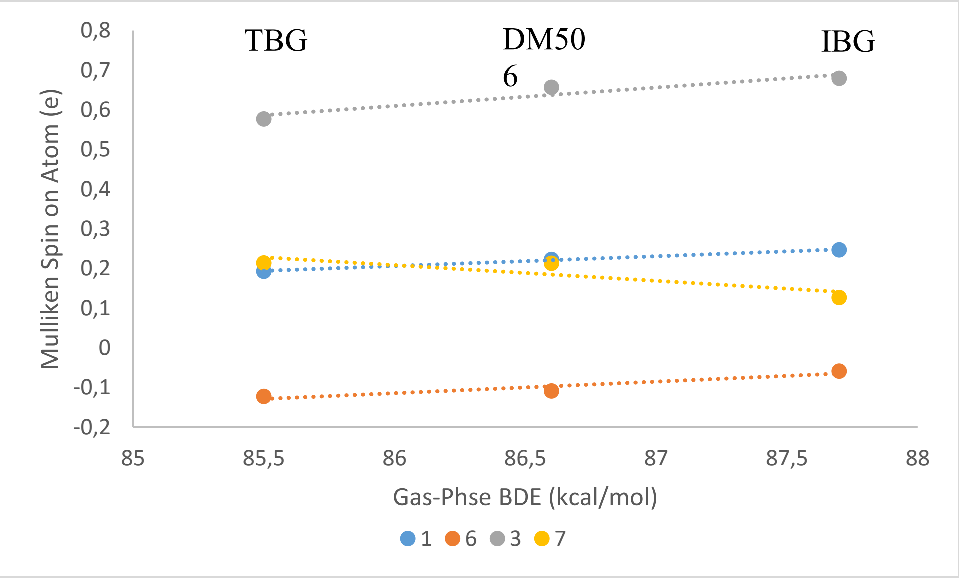

Figure S1. Plot of selected Mulliken spin densities for the radicals of DM506, TBG, and IBG formed from the scission of the N-H bond. Center numbers correspond to those in Table S1.

Figure S1 shows that the highest unpaired spin density for the (nominally) nitrogen-centered radicals of DM506, TBG, and IBG is located on the carbon labeled '3' in the five-membered ring moiety. In the main manuscript, this position is referred to as α to avoid confusion with the numbering convention used for the azepineindole skeleton. The carbon in the benzene moiety that is *para* to the nitrogen atom (labeled "6" in the structure in Table S1) shows little variation across the three compounds and is about one third of the spin density at "3". It stands to reason that the presence of an electron withdrawing group at "6" (as in IBG) will have little effect on the BDE as compared to DM506. Indeed, the effect of substituents on the stability of center "3", which holds

the majority of the unpaired spin density, would be expected to have the largest effect on BDEs. In TBG, the methoxyl group is *para* to position "3", indicating that it should lower the N-H BDE in this compound relative to DM506. BDE values from the *Comprehensive Handbook of Chemical Bond Energies*[1] indicate that the C–H bond dissociation energy in 4-methoxytoluene is 3.1 kcal/mol lower than that in toluene (89.7±1.2 kcal/mol), while the C–H BDE in 3-methoxytoluene is 0.8 kcal/mol higher. The latter suggests that the benzyl radical is preferentially stabilized by the OMe substituent when it is present at the *para* position and destabilized when it is present at the *meta* position. This supports the calculated trend in gas-phase BDEs for DM506, TBG and IBG presented in Figure S1.

Table S2. Molecular descriptors for ibogalogs and known antioxidants.

**Descriptor**	**DM506**	**TBG**	**IBG**	**PMHC**	**Stobadine**	**SMe1EC2**
LogP	2.5	2.6	2.6	2.6	1.7	2.4
LogBB	0.76	0.65	0.62	0.04	1.15	0.06
Vol ($Å^3$)	723	815	814	664	756	929
SASA ($Å^2$)	437	481	481	407	453	541
FOSA ($Å^2$)	235	323	324	217	317	349
FISA ($Å^2$)	24	29	33	57	24	85
PISA ($Å^2$)	178	122	121	132	111	107
PSA ($Å^2$)	19.0	29.2	29.2	31.3	20.6	66.1
Polarizability ($Å^3$)	24.9	26.9	26.9	21.0	25.2	30.8
Rotable bonds	0	1	1	1	0	2
Dipole moment	2.87	2.81	2.39	3.01	1.24	6.48
$Dipole^2$/Vol	0.011	0.010	0.007	0.014	0.002	0.045
Globularity	0.90	0.88	0.88	0.91	0.89	0.85
H-bond acceptor/donor	2/1	2.75/1	2.75/1	1.5/1	3/1	4.25/1
pK_a	10.3	10.3	10.3	N/A	N/A	N/A

Molecular descriptors were calculated using the QikProp software.

[1] Luo, Y.R., *Comprehensive Handbook of Chemical Bond Energies*. 2007: CRC Press.
